# Magnetic properties of monomeric and polymeric stannolediide yttrium and erbium complexes

**DOI:** 10.1038/s42004-025-01797-4

**Published:** 2025-11-21

**Authors:** Xiaofei Sun, Sören Schlittenhardt, Tingting Ruan, Masaichi Saito, Mario Ruben, Peter W. Roesky

**Affiliations:** 1 Institute for Inorganic Chemistry, Karlsruhe Institute of Technology (KIT), Karlsruhe, Germany; 2 Institute of Nanotechnology, Karlsruhe Institute of Technology (KIT), Eggenstein-Leopoldshafen, Germany; 3 Institute of Physical and Theoretical Chemistry, Goethe University Frankfurt, Frankfurt am Main, Germany; 4 Department of Chemistry, Graduate School of Science and Engineering, Saitama University, Saitama city, Saitama Japan; 5 Centre Européen de Science Quantique (CESQ), Institut de Science et d’Ingénierie Supramoléculaires (ISIS, UMR 7006), CNRS-Université de Strasbourg, Strasbourg Cedex, France; 6 Institute of Quantum Materials and Technologies (IQMT), Karlsruhe Institute of Technology (KIT), Eggenstein-Leopoldshafen, Germany

**Keywords:** Organometallic chemistry, Ligands, Magnetic properties and materials

## Abstract

Lanthanide sandwich complexes are promising candidates for single-molecule magnets, owing to their magnetic anisotropy and slow relaxation of magnetization. Here we show the synthesis and characterization of the polymeric yttrium and erbium complexes ligated by cyclooctatetraendiide and stannolediide ligands. The molecular structures showed the formation of one-dimensional-coordination polymers with the molecular formula of C_40_H_56_KOSi_2_SnLn (Ln = Y, Er), which resemble a trapezoidal wave. Upon encapsulation of the K cations with 2.2.2-cryptand, the respective monomeric sandwich complexes having the molecular formula of C_57_H_87_KN_2_O_6_Si_2_SnLn (Ln = Y, Er) were obtained. The diamagnetic yttrium complexes were characterized by NMR spectroscopy, and the magnetic properties of both Er complexes were studied. The polymeric Er complex demonstrates a lower energy barrier for magnetic relaxation compared to the monomeric erbium complex, despite the similar structural features of the compounds.

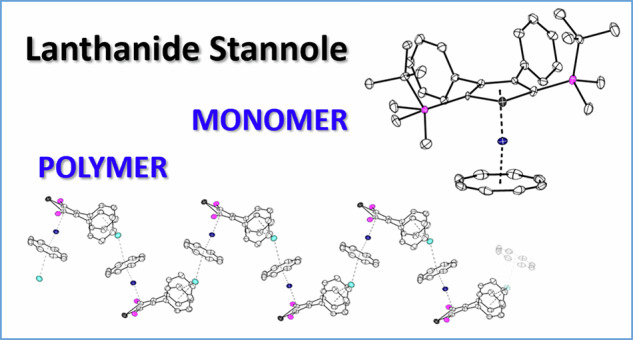

## Abstract (web summary)

Lanthanide sandwich complexes display interesting properties for diverse applications. Here, the authors synthesize monomeric and polymeric yttrium and erbium complexes ligated by cyclooctatetraendiide and stannolediide ligands and explore the magnetic properties of the erbium compounds.

## Introduction

Organometallic sandwich complexes are ubiquitous owing to their unique structure, diverse applications, and interesting properties. Among these complexes, a significant class of lanthanide sandwich complexes that exhibit unique electrical and magnetic properties features phthalocyanine-type ligands^1–5^. Another major group of lanthanide sandwich complexes is characterized by the use of carbocyclic π-ligands such as cyclopentadienide (Cp^−^) and cyclooctatetraendiide (COT^2^^−^) derivatives^6–10^. In the pursuit of further tuning the electronic properties of the cyclopentadienide, different five-membered heterocyclic ligands have been obtained through isolobal substitution of one C-R fragment with other heteroatom fragments. This has led to novel π-ligands with rich coordination chemistry, such as monoanionic group 15 heterocycles, namely, pyrrolide^11,12^, phospholide^13^, arsolide^14^, and bismolide^15^, and dianionic group 14 ligands including silolediide^16–18^, germolediide^19^, stanolediide^20^, and plumbolediide^21^. While rare-earth complexes incorporating group 15 heterocyclic anions have emerged in the late 1980s^22^, and have been well-documented for both trivalent and divalent rare-earth metals^23^, the utilization of dianionic group 14 ligands with rare-earth metals remains nascent, having been introduced only recently^24–31^. The introduction of the heavy group 14 element increases the flexibility of the ligand coordination, which gives the diverse chemistry due to the dianionic charge and the lone pair. The dianionic charge allows their utilization as *μ*–*η*^5^:*η*^5^ bridging π-ligand for the construction of multi-decker or polymeric species^32–34^, which is comparable to COT^2^^−^ ligands. In addition, the *η*^1^-coordination *via* the lone pair promotes dimensional expansion and facilitates the formation of macromolecular structure motifs^26^.

Single-molecule magnets (SMMs) with slow relaxation of magnetization represent a promising area for potential applications in high-density data storage and quantum applications. Lanthanide-based SMMs have become an emerging research area due to their high magnetic anisotropy and large magnetic moments^35^. Adjusting the crystal field and local symmetry around individual lanthanide ions have proven to be effective in raising the blocking temperature and effective energy barrier of SMMs^36^. For the prolate Er^3+^ ion, the interaction between the *J* = 15/2 ground state and the cyclooctatetraendiide (COT^2^^−^) crystal field induces a strong anisotropy axis. Therefore, the {Er(*η*^8^-COT)}^+^ unit has been used as a magnetic building block in the design of molecules that exhibit strong SMM behavior^37^. One rational approach to obtain such kind of efficient SMMs is to use [Er(*η*^8^-COT)I(thf)_3_]^38,39^ or [Er(*η*^8^-COT)BH_4_(thf)_2_]^40^ as a precursor and substitute the (pseudo)halogenide with other functional groups^28,37,39,41–50^.

To further maintain the linear sandwich structure motif of the Ln complexes and investigate the impact of introducing a soft Sn atom on the magnetic properties and solid-state structure, we studied the coordination chemistry of the stannolediide with suitable rare-earth COT-halogenide precursors.

## Results And Discussion

### Synthesis and structures

**Scheme 1:**
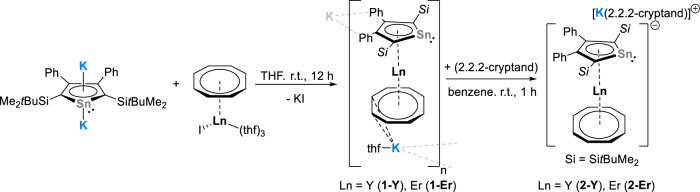
Synthesis of compounds **1-Ln** and **2-Ln**.

The target stannole-COT complexes [(*η*^8^-COT)Ln(*η*^5^-L^Sn^)K(thf)]_n_ (Ln = Y (**1-Y**), Er (**1-Er**), L^Sn^ = 1,4-bis-(*tert*-butyl-dimethylsilyl)-2,3-bis-phenyl-stannolediide) were synthesized by reacting [K_2_(Et_2_O)_0.45_(*η*^5^-L^Sn^)]^51^ with [Ln(*η*^8^-COT)I(thf)_3_]^38,39^ in THF at room temperature (Scheme 1). After stirring the reaction mixture for 12 h, the insoluble KI was removed by filtration. Single crystals of complexes **1-Y** and **1-Er** were obtained by layering the concentrated THF solution with *n*-pentane. The crystalline yields of **1-Y** and **1-Er** are 70% and 62%, respectively.

**Fig. 1:**
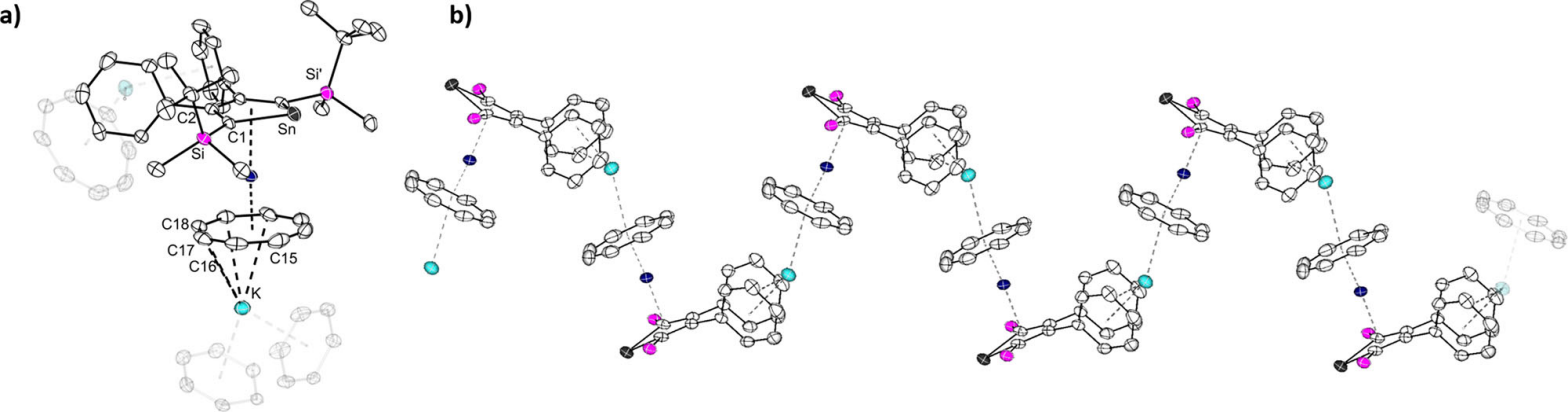
Molecular structure of the yttrium stannole complex 1-Y in the solid state. **a** Molecular structure of [(*η*^8^-COT)Y(*η*^5^-L^Sn^)K(thf)] in the solid state, the coordinated thf-molecule is omitted for clarity. **b** Section of the polymeric structure of **1-Y**. All structures are depicted with thermal ellipsoids at 40% level. The coordinated thf molecule, the Me and *t*Bu groups on the silyl substituent are omitted for clarity. All hydrogens are also omitted. Selected bond lengths and bond angles are summarized in Table 1 and the ESI. Color code: Y, dark blue; K, cyan; Sn, grey; Si, pink; C, white.

The molecular structures of **1-Ln** were determined by single-crystal X-ray diffraction analysis. They are isostructural and crystallized as one-dimensional coordination polymers in the orthorhombic space group *Pnma*. The asymmetric units of **1-Ln** contain half of the [(*η*^8^-COT)Ln(*η*^5^-L^Sn^)K(thf)] molecule, with Ln, K, and Sn on a crystallographic mirror plane. Figure 1 shows the molecular structure of **1-Y** and a section of the coordination polymer. The macromolecular structure bears resemblance to a trapezoidal wave, similar lanthanide-based coordination polymers were obtained for example, in the homoleptic lanthanide multidecker species with bulky COT ligands^42^. As expected, the Ln^3+^ metal ions are sandwiched between the *η*^5^-stannole and *η*^8^-COT ligands in nearly linear metallocene-type sandwich motifs with Ct_Cot_–Ln–Ct_Sn_ (Ct = centroid of the corresponding ring) angles of 176.3° (**1-Y**) and 176.5° (**1-Er**). In the yttrium complex **1-Y**, the Y–C distances to the COT ring are found to be between 2.564(7) and 2.594(7) Å, and the distance from Y to the centroid of the COT ring was found to be 1.816 Å (Table 1). For the erbium congener, the corresponding values are marginally shorter due to the slightly smaller ionic radius of Er^3+^ compared with Y^3+^. A similar trend was also observed for the corresponding Ct_Sn_–Ln distances, which were found to be 2.303 Å (**1-Y**) and 2.274 Å (**1-Er**), respectively. The Ln–Sn distances are 3.1336(11) Å (**1-Y**) and 3.1081(1) Å (**1-Er**). The K cation is located on the other side of the COT ring, with notable discrepancies in the K-C_COT_ distances ranging from 3.085(7) Å (K–C18) to 3.6669(1) Å (K···C15) in **1-Y** and from 3.100(5) Å (K–C18) to 3.6418(1) Å (K···C15) in **1-Er**. Therefore, the COT ring is formally *η*^4^-coordinated (C17, C17’, C18, C18’) to the K^+^ ions. The K^+^ ions are further coordinated by secondary π-interaction from the phenyl groups of the neighboring sandwich fragments, thus resulting in the formation of the infinite polymeric chain. Interestingly, the polymeric structure is different as compared to the coordination polymer observed for heteroleptic germole-COT species^28^. As in the latter case, the coordination polymer to a first approximation a linear chain, with both the Ln^3+^ and K^+^ being sandwiched between the *η*^8^-COT and *η*^5^-germole. Within the stannole ring, the existence of the mirror plane indicates the presence of π-electron delocalization, which is further corroborated by the nearly planar five-membered rings, as the sum of the internal angles is close to 540° (Table 1).

**Table 1:** Selected bond lengths and -angles of compounds 1-Ln and 2-Ln

Compound	1-Y	1-Er	2-Y	2-Er
Ln–Ct_COT_ [Å]	1.8156	1.789	1.797	1.768
Ln–Ct_Sn_ [Å]	2.303	2.274	2.301	2.280
K–Ct_COT_ [Å]	2.846	2.835		
Ct_Cot_–Ln–Ct_Sn_ [°]	176.3	176.5	167.3	168.9
Sum of internal angle within the stannole ring [°]	537.914	538.102	538.481	538.463

**Fig. 2:**
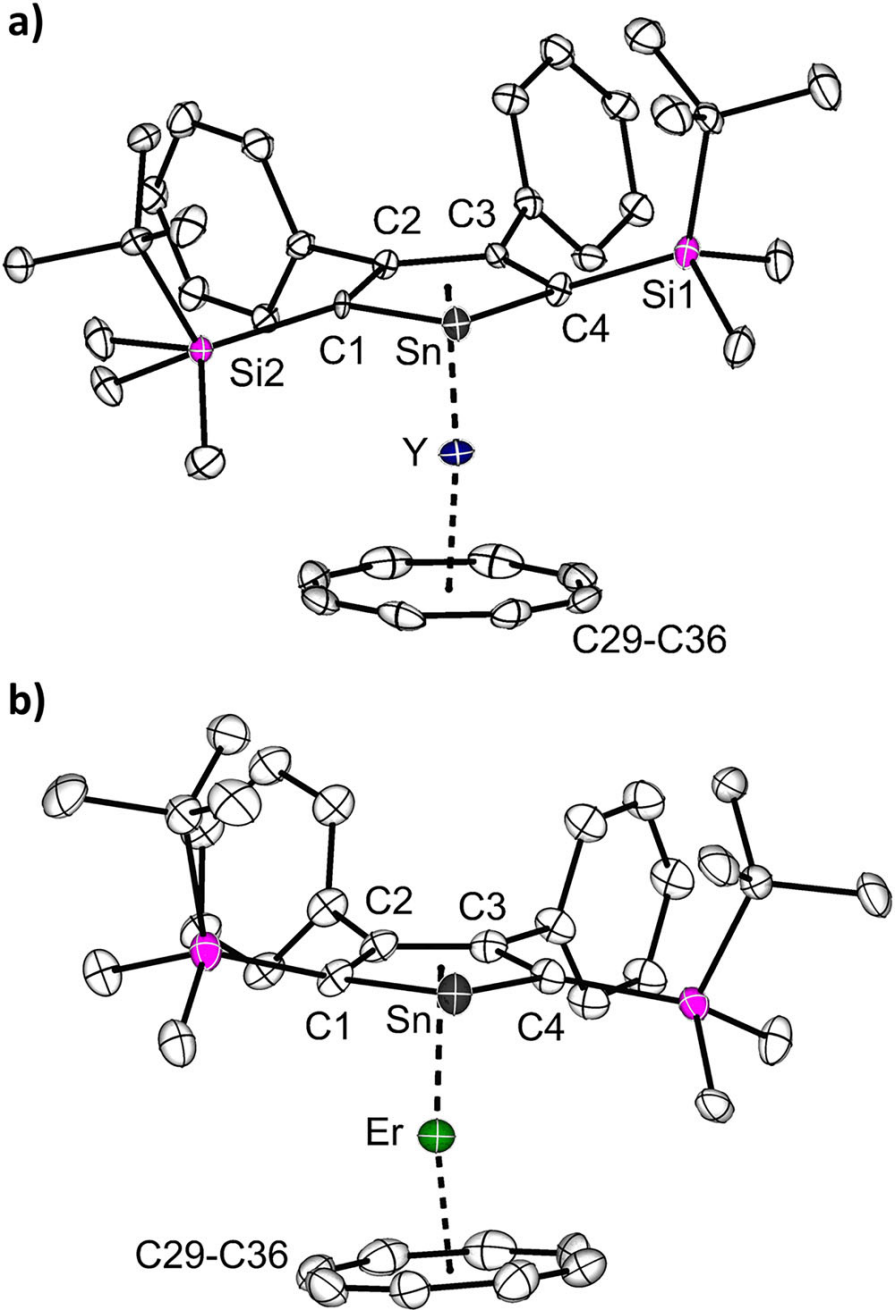
Molecular structures of the monomeric stannole complexes. **a** The structure of the yttrium complex **2-Y**. **b** The structure of the erbium complex **2-Er**. For both structures, only the anionic part of the structure is depicted. The structures are depicted with thermal ellipsoids at 40% level. All hydrogens are omitted for clarity. Selected bond lengths and bond angles are summarized in Table 1 and the ESI. Color code: Er, green; Y, dark blue; Sn, grey; Si, pink; C, white.

The addition of [2.2.2]-cryptand to the polymeric **1-Ln** in benzene led to the formation of the charge-separated monomeric sandwich compounds [(*η*^8^-COT)Ln(*η*^5^-L^Sn^)][K(2.2.2)-crypt] (Ln = Y (**2-Y**), Er (**2-Er**)). After leaving the suspension stand for *ca*. 1 h at room temperature, orange crystalline samples of **2-Ln** formed. While preparing this manuscript, the group of Layfield disclosed the related compound [(*η*^8^-COT)Er(*η*^5^-L^Sn’^)][K(2.2.2)-crypt] (L^Sn’^ = {SnC_4_-2,5-(SiMe_3_)_2_-3,4-Me_2_}^2–^)^29^. Crystals of **2-Y** and **2-Er** were obtained in yields of 85% and 71%, respectively. Both molecular structures were determined by single-crystal X-ray diffraction analysis, and they crystallize in the monoclinic space group *P*2_1_/*m*. The structures are very similar, both consisting of an ion pair, with the cationic part being the cryptand-chelated K^+^ cation. The anionic part [(*η*^8^-COT)Ln(*η*^5^-L^Sn^)]^−^ is as expected, essentially a slightly bent monomeric sandwich structure (Fig. 2), reminiscent of the parent carbon-based [(*η*^8^-COT)Ln(*η*^5^-Cp*)]^52^ and other recently reported heteroleptic COT double-decker compounds with germole^28,31^, stannole^29,30^, plumbole^25^, phosphole^43^, and arsole^53^. Upon decoordination of the K^+^ ion, the sandwich motif deviates slightly more from linearity (Ct_Cot_–Ln–Ct_Sn_ 167.3 for **2-Y** and 168.9° for **2-Er**) than in their polymeric analogs **1-Y** and **1-Er**. Another consequence of the removal of K^+^ is the decrease in the Ln–COT distances of about 0.02 Å in both structures (Table 1). When compared with other erbium sandwich complexes comprising the unsubstituted COT ligand, the Er-Ct_COT_ distance of 1.768 Å is shorter than the values in the homoleptic [Er(*η*^8^-COT)_2_]^−^ (1.8744(3) Å)^49,54^ but comparable to the recently reported [(*η*^8^-COT)Er(*η*^5^-germole)]^−^ (1.874 Å)^28^ and slightly longer than in the corresponding heteroleptic COT compounds with pentamethylcyclopentadienide (1.727 Å)^52^, phosphole (1.686 Å)^43^ and arsole (1.677-1.705 Å)^53^. The distances between the stannole ring and the Ln^3+^ is almost not affected. As expected the structural parameters are similar to Layfields [(*η*^8^-COT)Er(*η*^5^-L^Sn’^)][K(2.2.2)-crypt]^29^. It is noteworthy to mention that in the molecular structures of **1-Er** and **2-Er**, the shortest Er···Er separations are 8.4 (**1-Er**) and 9.6 Å (**2-Er**), indicating minimal dipole-dipole interactions.

The yttrium complexes **1-Y** and **2-Y** were further analyzed NMR spectroscopically. The ^1^H NMR spectra recorded in THF-*d*_8_ of both compounds show great similarities, with three singlet signals of the silyl group at *δ* = −0.36, 0.39, and 0.69 ppm in 1:1:3 molar ratio. The eight protons of the COT ring were detected as a singlet signal at *δ* = 6.28 ppm for **1-Y** and 6.27 ppm for **2-Y**, showing their equivalency in solution. Therefore, the molecular structures of the double-decker motifs exhibit *C*_s_ symmetry to a first approximation in THF solution. In the ^29^Si{^1^H} NMR spectrum, one singlet at *δ* = −3.6 ppm is observed for both compounds. The ^89^Y NMR spectrum shows one resonance at *δ* = −16.7 ppm and −18.6 ppm for **1-Y** and **2-Y**. In both cases, the ^119^Sn{^1^H} signals (*δ* = 779.4 ppm for **1-Y** and 774.7 ppm for **2-Y**) are shifted to higher frequencies compared to the starting material [K_2_(Et_2_O)0.45(*η*^5^-L^Sn^)] (*δ* = 615.5 ppm)^51^. The good solubility of **1-Y**, together with the similar sharp and well-defined signals observed in the NMR spectra of **1-Y**, suggests that it adopts a monomeric structure in solution, which is similar to **2-Y**.

### Magnetic properties

As previous reports as well as theoretical studies have shown that Er(III) complexes of the form COT-Er-L are typically observed to exhibit slow magnetic relaxation^25,28,43,53,55^, we have investigated the magnetic properties of the two Er(III) containing complexes **1-Er** and **2-Er**. As the structural properties of **2-Er** are very comparable to those of [(*η*^8^-COT)Er(*η*^5^-L^Sn’^)][K(2.2.2)-crypt] recently reported by Layfield and coworkes^29^, only differing in the substituents of the stannole ligand, this offers a rare opportunity to investigate the influence of those on the magnetic properties.

We have performed temperature and field-dependent DC measurements on **1-Er** and **2-Er**. The *T*-dependence was measured under an applied magnetic field of 1000 Oe upon cooling down the samples in a temperature range of 300 K–2 K (Fig. S20, S24). We have obtained room temperature *χT* values of 11.65 and 11.91 cm^3 ^K mol^-1^, for **1-Er** and **2-Er** respectively. Both values are in good agreement with the theoretically expected value of 11.48 cm^3 ^K mol^−^^1^ for a single uncoupled Er(III) ion. Upon cooling, the experimental susceptibility data of both compounds gradually decrease to reach final values of 8.56 cm^3 ^K mol^−^^1^, for both **1-Er** and **2-Er** at 2 K. The temperature dependence of both compounds is nearly identical, indicating that Er-Er interactions within the polymeric structure of **1-Er** are negligible at first glance. At low temperatures the field dependencies of the magnetization have been recorded between 0 and 7 T. Again, both compounds reveal near identical behavior reaching values close to 5 *μ*_*B*_ at 7 T. The slightly higher value compared to 4.5 *μ*_B_ is often observed in compounds with significant anisotropy.

Recording hysteresis loops with a magnetic sweep rate of 20 Oe/s and 100 Oe/s, both compounds show waist-restricted open loops (Figs. S21, S22, S25), which is typical for such COT-Er-L systems due to efficient quantum tunnelling around zero-field. In both cases, the loops are closing around 6 K. The previously mentioned COT-Er-stannole complex of Layfield and coworkes^29^ was similarly reported with open loops up until about 6 K. It should be noted that the hysteresis measurements in their case were performed using a sweep rate of 200 Oe/s.

**Fig. 3:**
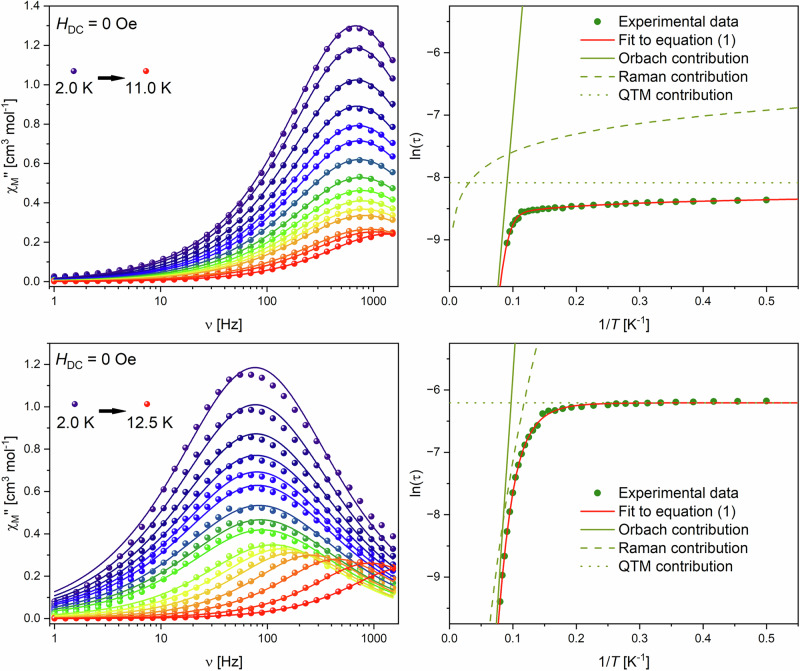
Magnetic measurements. Frequency-dependent out-of-phase component of the magnetic susceptibility (left) and temperature dependence of the magnetic relaxation times (right) for **1-Er** (top) and **2-Er** (bottom). The solid lines (left) are the best fits to a generalized Debye model.

Measurements of the AC magnetic susceptibility are the first measurements, which clearly show differences between **1-Er** and **2-Er**. Both compounds reveal a frequency-dependent maximum in the out-of-phase component (*χ“*) of the susceptibility. While the maximum at the lowest observed temperature of 2 K is centered around 70 - 80 Hz for the monomer **2-Er**, the polymer **1-Er** exhibits its maximum at a considerably higher frequency of about 700−800 Hz. In both cases upon increasing the temperature the maximum decreases in intensity, starts shifting to higher frequencies and eventually is outside of our detectable window at 11.0 K and 12.5 K for **1-Er** and **2-Er**, respectively (Fig. 3). Using a generalized Debye model to extract the experimental relaxations times and fitting their temperature dependence using:

1 $${\tau }^{-1}={\tau 0}^{-1}\exp (-{U}_{{eff}}/{k}_{B}T)+C{T}^{n}+{\tau {QTM}}^{-1}$$

we were able to get deeper insight into the relaxation behavior.

For both datasets we were able to obtain good fits to the described model yielding the following parameters: τ_0_ = 7.97 × 10^−^^9^ s, *U*_eff_ = 81 ± 6 cm^−^^1^, C = 760.33 K^−^^n^ s^−^^1^, n = 0.42 and τ_QTM_ = 3.08 × 10^−^^4 ^s for the polymer **1-Er** and τ_0_ = 7.07 × 10^−^^10^ s, *U*_eff_ = 106 ± 9 cm^−^^1^, C = 2.14 × 10^−^^3 ^K^−^^n^ s^−^^1^, *n* = 5.77 and τ_QTM_ = 2.02 × 10^−^^3 ^s for the monomer **2-Er** (Table 2). The monomeric structure **2-Er** was compared with the reported complex [(*η*^8^-COT)Er(*η*^5^-L^Sn’^)][K(2.2.2)-crypt] (Table 2). Despite their very similar structural appearance the obtained *U*_eff_ value of our system is with 106 cm^-1^ about 20 cm^−^^1^ lower than the 125 cm^−^^1^ reported for [(*η*^8^-COT)Er(*η*^5^-L^Sn’^)][K(2.2.2)-crypt]. Differences for the obtained energy barrier in such erbium-based systems are often observed due to differences in their structural parameters, especially the distance between the central ion and the aromatic ligands Er–Ct_COT_ and Er–Ct_Sn_, where a short Er–Ct_COT_ distance is known to enhance SMM performance and vice versa for Er–Ct_Sn_. In case of **2-Er** and [(*η*^8^-COT)Er(*η*^5^-L^Sn’^)][K(2.2.2)-crypt] the Er–Ct distances obtained through X-ray crystallography are the same within the error range. Synthetically the difference between **2-Er** and [(*η*^8^-COT)Er(*η*^5^-L^Sn’^)][K(2.2.2)-crypt] are the phenyl and *tert-*butyl-dimethylsilyl groups in **2-Er** compared to methyl and TMS in [(*η*^8^-COT)Er(*η*^5^-L^Sn’^)][K(2.2.2)-crypt]_._ The two factors that influence the relaxation behavior are, therefore, the different electronic densities of the stannole ring due to more electron rich substituents as well as the steric effect which causes a lowered Ct–Er–Ct angle of 169° in **2-Er** compared to 176° in [(*η*^8^-COT)Er(*η*^5^-L^Sn’^)][K(2.2.2)-crypt]_._ Previous studies have shown that the Ct–Er–Ct angle is a highly relevant measure in such systems^55^. Further, the more electron donating substituents increase the electronic density on the stannole ring and, therefore, increase the effect of the non-beneficial axial ligand field provided. We further validate this assumption by the energy barrier of 81 cm^-1^ determined for the polymeric structure **1-Er**. From crystallographic analysis, the structural parameters of the Er–Ct_COT_ and Er–Ct_Sn_ distances as well as the Ct–Er–Ct angle are nearly identical between **1-Er** and [(*η*^8^-COT)Er(*η*^5^-L^Sn’^)][K(2.2.2)-crypt]_._ However, the relaxation barrier found for **1-Er** is even lower than that of **2-Er** which is, therefore, most likely a result of the changed electronic densities due to the potassium cation within the coordination polymer.

**Table 2:** Relaxation parameters obtained from AC susceptibility measurements for 1-Er, 2-Er and [(*η*^8^-COT)Er(*η*^5^-L^Sn’^)][K(2.2.2)-crypt]^29^

Compound	1-Er (this work)	2-Er (this work)	[(*η*^8^-COT)Er(*η*^5^-L^Sn’^)][K(2.2.2)-crypt]
*τ*_*0*_ [s]	7.97 × 10^−^^9^	7.07 × 10^−^^10^	1.14 × 10^−^^9^
*U*_eff_ [cm^-1^]	81 ± 6	106 ± 9	125 ± 3
*C* [K^-n^ s^-1^]	760.33	2.14 ×10^-3^	0.57
*n* [·]	0.42	5.77	2.4
*τ*_*QTM*_ [s]	3.08 × 10^−^^4^	2.02 × 10^−^^3^	1.8 × 10^−^^2^

### Ab initio calculations

**Fig. 4:**
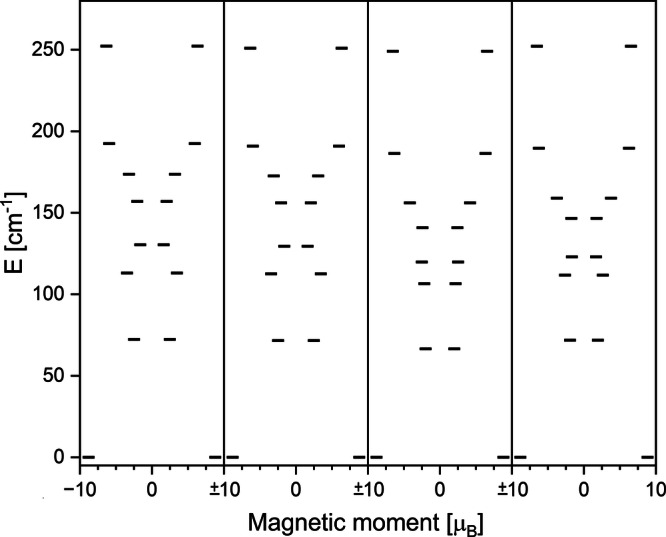
Ab initio calculations. Ab initio energy diagram of the crystal field splitting of the eight lowest lying KD states for **2-Er**, **2-Er**^**TMS**^, **2-Er**^**tBu**^ and **2-Er**^**Me**^ (left to right).

In order to get a better understanding of the relaxation mechanism we have performed multireference CASSCF/RASSI/SINGLE_ANISO calculations using the OpenMolcas software package^56^. Due to high computational effort for the complete polymeric structure of **1-Er** we opted to exchange the *tert*-butyl-dimethylsilyl with methyl groups and calculate a small fragment of the type COT-Y-L^Sn^-K-COT-Er-L^Sn^-K-COT-Y-L^Sn^ (**1-M**^**Me**^). As the change from a large substituent like *tert*-butyl-dimethylsilyl to methyl appears drastic, we have performed calculations of **2-Er**, and three fictional molecules **2-Er**^**TMS**^, **2-Er**^**tBu**^, and **2-Er**^**Me**^ in which the size of the two *tert*-butyl-dimethylsilyl is successively decreased to TMS, *tert-*butyl, and finally methyl. Note that we have kept the bond lengths and tilting angles similar across this series, so that the observed difference can be fully ascribed to electronic effects. Figure 4 shows the obtained energy diagram of the eight lowest Kramers doublets (KDs) within the *J* = 15/2 multiplet for **2-Er,** **2-Er**^**TMS**^, **2-Er**^**tBu**^ and **2-Er**^**Me**^.

**Fig. 5:**
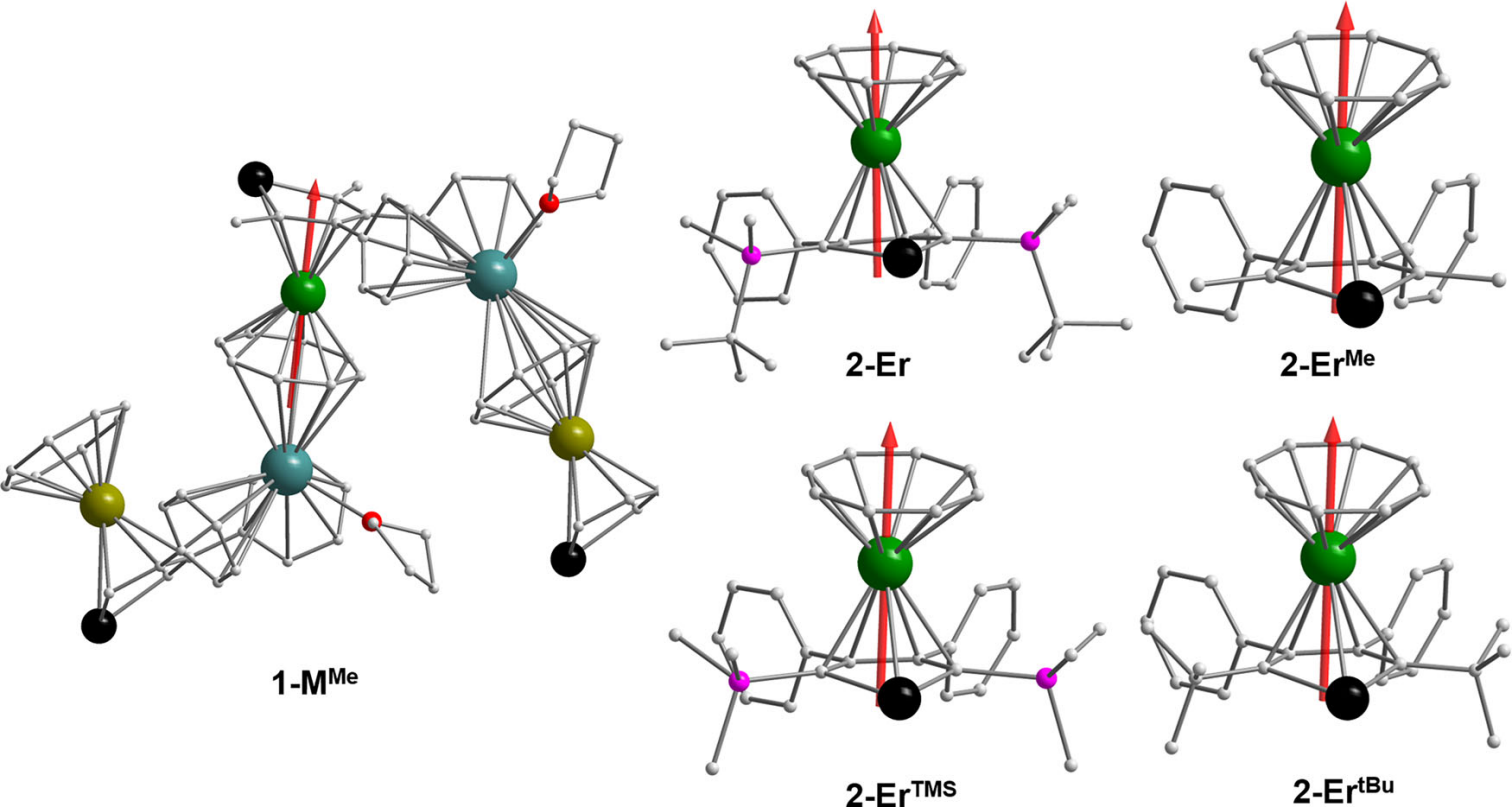
Computed structures with their magnetic easy axis.

Upon changing from *tert*-butyl-dimethylsilyl to TMS the energy diagram remains essentially unchanged, which is expected due to the fact that the change occurs far away from the Er ion. The calculated energy of the first excited state for **2-Er**^**TMS**^ and **2-Er** is 72 cm^−^^1^ in both cases (Table S4 and S5). The different strengths of the electron donating effect of the *tert*-butyl-dimethylsilyl- and TMS-group towards the stannole moiety is negligible, and because of that, practically no change is observed. More interestingly, for **2-Er**^**tBu**^, a small but noticeable change is observed, which can be fully attributed to the stronger inductive effect of the *tert-*butyl group (Table S6). Since the axial ligand field of the stannole ligand is non-preferable for stabilizing the anisotropic character of Er(III), the increased electron density leads to the decrease in the energies of the excited KD states. In numbers, the energy of the first excited KD for **2-Er**^**tBu**^ is found at 66 cm^−^^1^, about 6 cm^−^^1^ lower than that of **2-Er**. The result of the last model compound **2-Er**^**Me**^ is again much closer to that of the complete structure **2-Er**, with smaller changes in the excited state energies (Table S7). However, towards higher excited states TMS holds as the better approximation. In all four cases the magnetic easy axis is aligned closely to the center of the COT- and L^Sn^-rings (Fig. 5). Based on these findings we conclude that exchanging bulky substituents like the *tert*-butyl-dimethylsilyl group with methyl groups is a valid approach for ab initio calculations with lower computational demand. It is further a good estimate of how strongly the inductive effects of substituents play a role for SMM performance in comparison to the structural changes invoked by the bulkiness. It appears that even drastically appearing changes do not influence the resulting energy diagram substantially. In fact, the biggest calculated energy change we were able to observe by exchanging substituents was about 6 cm^−^^1^, which is so small that it might not even be detectable, given common experimental errors of the relaxation barriers are within a few wavenumbers. Therefore, the influence of substituents of heterocyclic ligands on the energy diagram of lanthanide ions seems to be almost entirely due to the structural changes (bond lengths and angles) they invoke rather than changes in the ligand’s electron density.

**Fig. 6:**
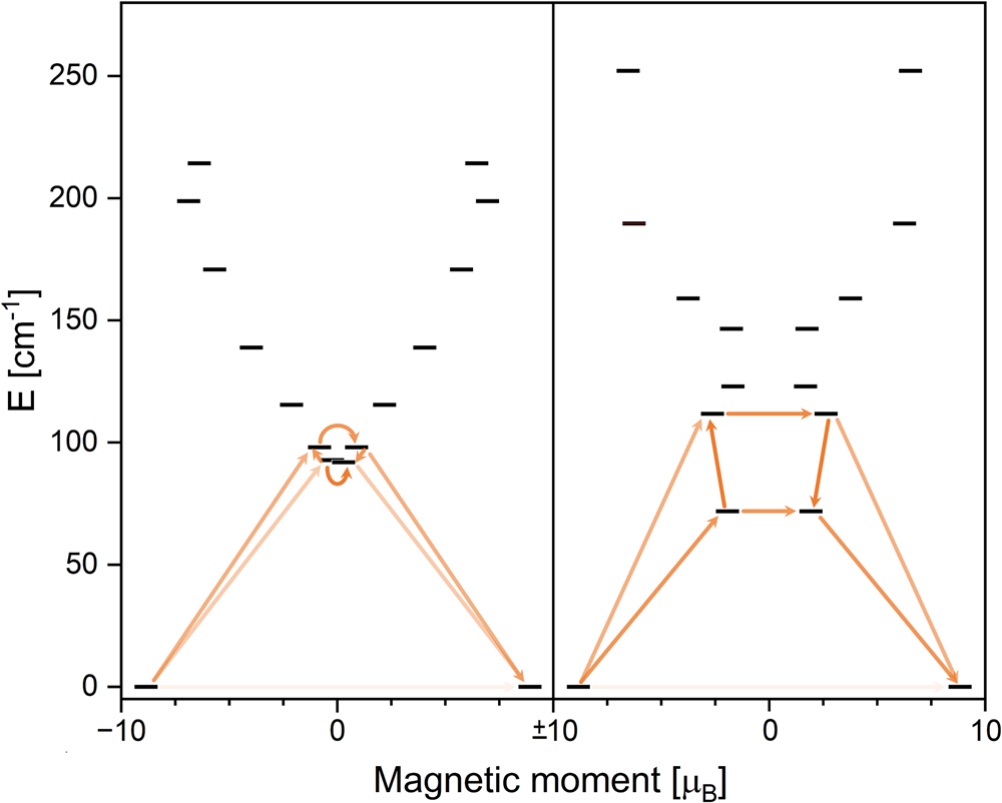
Ab initio calculations. Ab initio energy diagram and proposed relaxation pathway based on calculated transition probabilities for **1-Er** (left) and **2-Er** (right). Lighter shadings of the arrows indicate a low, darker shadings a high transition probability.

With this in mind, the comparison hereafter between **1-Er** and **2-Er** is based on the computational results of the structures where *tert*-butyl-dimethylsilyl was replaced with methyl. In both structures, the calculated ground state doublets have a strongly axial g-tensor with *g*_*x*_ = 0.0052, *g*_*y*_ = 0.0065, and *g*_*z*_ = 17.78 for **1-Er** and *g*_*x*_ = 0.0142, *g*_*y*_ = 0.0249, and *g*_*z*_ = 17.68 for **2-Er**. The wavefunctions in both cases correspond to *m*_*J*_ = 15/2 with only very small admixing of other wavefunctions. The energies of the first excited KDs are 92 cm^−^^1^ and 72 cm^−^^1^ for **1-Er** and **2-Er**, respectively, with the wavefunctions showing strong mixing. Interestingly, this would suggest that the energy barrier of magnetic relaxation of **1-Er** is higher than that of **2-Er**, which contradicts our experimental findings. However, investigation of the transition probabilities between the states reveals that the relaxation in **1-Er** is mainly mediated via the first excited doublet state where the spin flip is about ten times more efficient than further excitation towards the second excited doublet. Nevertheless, the transition probabilities between the ground state and the second excited state are not fully negligible and contributions of relaxation via the second excited doublet are possible (Fig. 6). In contrast, the transition probabilities for **2-Er** between the first and second excited state are even favorable to the spin flip within the first excited doublet, suggesting that there is a major contribution of the second excited KD to the relaxation mechanism of **2-Er** (Fig. 6).

The energies found for the second excited KDs are 98 cm^−^^1^ and 112 cm^−^^1^ for **1-Er** and **2-Er**, respectively. The combination of the excited state energies and the transition probabilities results in a good agreement with our experiment, as it explains a higher energy barrier for **2-Er**. Similar to the four calculated monomeric compounds, the magnetic easy axis in **1-Er** is found along the centers of the ligand (Fig. 5). Layfield and coworkers have also reported SINGLE_ANISO results for their compound [(*η*^8^-COT)Er(*η*^5^-L^Sn’^)][K(2.2.2)-crypt]^29^. Interestingly, the total crystal field splitting in their case was calculated as 253 cm^−^^1^, which perfectly matches that of our monomeric compound **2-Er** at 252 cm^−^^1^. However, the energy gap towards the early excited states, which in this type of complex is the metric yielding the effective barrier, significantly differs between the two compounds: 135 cm^−^^1^ for [(*η*^8^-COT)Er(*η*^5^-L^Sn’^)][K(2.2.2)-crypt] and 72 cm^−^^1^ for **2-Er**. As described above, the difference between the TMS group in [(*η*^8^-COT)Er(*η*^5^-L^Sn’^)][K(2.2.2)-crypt] and the *tert*-butyl-dimethylsilyl group in **2-Er** is negligible, and the differences between the two systems are attributed to the phenyl groups, especially to the invoked change of the Ct-Er-Ct angle due to the steric effect.

## Conclusion

In conclusion, we provided the synthetic approach on stannolediide-ligated rare-earth complexes, which feature one-dimensional coordination polymers in the solid state. Upon reaction with 2.2.2-cryptand, the polymeric complexes were converted into the naked monomeric anionic sandwich complexes. The erbium-based polymeric (**1-Er**) and monomeric (**2-Er**) stannolediide complexes exhibit differences in their magnetic properties. The polymeric complex **1-Er** demonstrates a lower energy barrier for magnetic relaxation compared to the monomeric **2-Er**, despite the similar structural features of the two systems. This difference in behavior can be attributed to the coordination environment, particularly the role of the potassium ion in the polymer. Furthermore, a comparison with the previously reported Er-COT-stannole complex, [(*η*^8^-COT)Er(*η*^5^-L^Sn’^)][K(2.2.2)-crypt], reveals that the energy barrier for magnetic relaxation of **2-Er** is slightly lower than that of [(*η*^8^-COT)Er(*η*^5^-L^Sn’^)][K(2.2.2)-crypt]. Despite the structural similarities. A computational study of several model complexes reveals that the inductive effect of the substituents on the heterocyclic ligand is almost fully negligible, and changes in the bonding angles play a more significant role. Nonetheless, calculations of **1-Er** do confirm that the impact of the potassium cation is highly relevant. These insights provide valuable information for the design of future lanthanide-based SMMs, where both electronic and steric factors play a crucial role in modifying their magnetic properties.

## Methods

All air- and moisture-sensitive manipulations were performed under dry N_2_ or Ar atmosphere using standard Schlenk techniques or in an argon-filled MBraun glovebox, unless otherwise stated. *n*-pentane and toluene were dried using an MBraun solvent purification system (SPS-800) and degassed. THF and benzene were distilled under nitrogen from potassium benzophenone ketyl. THF-*d*_8_ was dried over Na-K alloy and degassed by freeze-pump-thaw cycles. [K_2_(Et_2_O)_0.45_(*η*^5^-L^Sn^)]^51^ and [Ln(*η*^8^-COT)I(thf)_3_]^38^ were prepared according to the literature procedures. All other chemicals were obtained from commercial sources and used without further purification. Elemental analyses were carried out with an Elementar vario MICRO cube. NMR spectra were recorded on Bruker spectrometers (Avance Neo 300 MHz, Avance Neo 400 MHz or Avance III 400 MHz). Chemical shifts are referenced internally using signals of the residual protio solvent (^1^H) or the solvent (^13^C{^1^H}) and are reported relative to tetramethylsilane (^1^H, ^13^C{^1^H}), or externally relative to tetramethylsilane (^29^Si), tetramethyltin (^119^Sn). All NMR spectra were measured at 298 K, unless otherwise specified. The multiplicity of the signals is indicated as s = singlet, d = doublet, dd = doublet of doublets, t = triplet, q = quartet, m = multiplet and br = broad. Assignments were determined based on unambiguous chemical shifts, coupling patterns and ^13^C-DEPT experiments or 2D correlations (^1^H-^1^H COS, ^1^H-^13^C HMQC and ^1^H-^13^C HMBC). Infrared (IR) spectra were recorded in the region 4000–400 cm^−^^1^ on a Bruker Tensor 37 FTIR spectrometer equipped with a room temperature DLaTGS detector, a diamond attenuated total reflection (ATR) unit, and a nitrogen-flushed chamber. In terms of their intensity, the signals were classified into different categories (vs = very strong, s = strong, m = medium, w = weak, and sh = shoulder).

### Synthesis of complex 1-Y

To a mixture of [K_2_(Et_2_O)_0.45_(*η*^5^-L^Sn^)] (100.0 mg, 0.150 mmol) and [(COT)YI(thf)_3_] (80.9 mg, 0.150 mmol) was added 10 mL of THF at room temperature. After stirring the reaction mixture for 12 h at room temperature, the solution was filtered to remove the metathesis salt KI. The remaining dark red solution was concentrated and layered with *n*-pentane to form complex **1-Y** as red single crystals. Crystalline yield: 89.1 mg (70%). Anal. Calcd for C_36_H_48_KSi_2_SnY 0.85 (C_4_H_8_O) (844.96): C 55.01; H 6.54. Found: C 55.31, H 6.61. ^1^H NMR (400 MHz, THF-*d*_8_): *δ* (ppm) = 6.92–6.67 (m, 10H, C*H*_Ph_), 6.28 (s, 8H, C*H*_COT_), 3.63–3.60 (*ca*. 0.85 *thf*), 1.79–1.76 (*ca*. 0.85 *thf*), 0.69 (s, 18H, Si*tBu*Me_2_), 0.39 (s, 6H, Si*t*Bu*Me*_2_), −0.36 (s, 6H, Si*t*Bu*Me*_2_). ^13^C{^1^H} NMR (101 MHz, THF-*d*_8_): *δ* (ppm) = 173.9 (d, *J*_C-_ = 5.3 Hz, *C*_a_), 152.1 (*C*_b_), 151.3 (*C*_Ph_), 125.8 (*C*H_Ph_), 124.0 (*C*H_Ph_), 92.6 (d, *J*_C-_ = 2.6 Hz, *C*H_COT_), 68.4 (*C*H_2 thf_), 29.8 (SiC(*C*H_3_)_3_Me_2_), 26.6 (*C*H_2 thf_), 18.2 (Si*C*(CH_3_)_3_Me_2_), 5.7 (Si*t*Bu*Me*_2_), 0.8 (Si*t*Bu*Me*_2_). One *C*H_Ph_ signal is missing. ^29^Si{^1^H} NMR (80 MHz, THF-*d*_8_): *δ* (ppm) = -3.6. ^119^Sn{^1^H} NMR (80 MHz, THF-*d*_8_): *δ* (ppm) = 779.4. ^89^Y NMR (15 MHz, THF-*d*_8_): *δ* (ppm) = –16.7. IR (ATR): $\widetilde{\nu }\,$(cm^−^^1^) = 3078 (sh), 3056 (w), 3021 (w), 2950 (vs), 2926 (vs), 2883 (s), 2850 (vs), 2705 (w), 2647 (w), 2323 (w), 2288 (w), 2165 (w), 2115 (w), 2079 (w), 2040 (w), 1983 (w), 1591 (m), 1556 (sh), 1466 (s), 1439 (m), 1406 (w), 1388 (w), 1359 (w), 1311 (m), 1245 (s), 1185 (w), 1070 (w), 1050 (w), 1005 (w), 961 (s), 914 (w), 894 (w), 819 (sh), 803 (vs), 771 (s), 700 (s), 670 (sh), 651 (sh), 625 (sh), 488 (w), 446 (w), 414 (w).

### Synthesis of complex 1-Er

To a mixture of [K_2_(Et_2_O)_0.45_(*η*^5^-L^Sn^)] (100.0 mg, 0.150 mmol) and [Er(COT)I(thf)_3_] (92.7 mg, 0.150 mmol) was added 10 mL of THF at room temperature. After stirring the reaction mixture for 12 h at room temperature, the solution was filtered to remove the metathesis salt KI. The remaining dark red solution was concentrated and layered with *n*-pentane to form complex 1-Er as red single crystals. Crystalline yield: 86.9 mg (62%). Anal. Calcd for C_36_H_48_KSi_2_SnEr (C_4_H_8_O) (934.12): C 51.43; H 6.04. Found: C 51.71, H 6.01. IR (ATR): $\widetilde{\nu }\,$(cm^−^^1^) = 3047 (w), 3011 (w), 2947 (vs), 2923 (vs), 2877 (m), 2846 (vs), 2760 (w), 2728 (w), 2700 (w), 2324 (w), 2166 (w), 1983 (w), 1886 (w), 1853 (w), 1749 (w), 1590 (m), 1568 (w), 1553 (m), 1490 (m), 1466 (m), 1438 (w), 1401 (w), 1384 (w), 1387 (w), 1312 (m), 1237 (m), 1185 (m), 1070 (m), 1050 (m), 1028 (w), 1005 (w), 958 (s), 914 (w), 895 (m), 866 (w), 819 (s), 798 (vs), 767 (m), 714 (sh), 705 (vs), 671 (sh), 651 (m), 626 (w), 589 (m), 487 (w), 450 (w), 414 (m).

### Synthesis of complex 2-Y

To a mixture of complex **1-Y** (40.0 mg, 0.047 mmol) and [2.2.2]-cryptand (22.6 mg, 0.60 mmol) was added ca. 1 mL of benzene, and the suspension was kept at room temperature for 1 h. Red-colored single crystals were formed, and the remaining solution was removed carefully with a syringe. The crystals were washed twice with ca. 2 ml of *n*-hexane to remove the unreacted [2.2.2]-cryptand and dried under vacuum. Crystalline yield: 46.3 mg (85%). Anal. Calcd for C_54_H_84_KN_2_O_6_Si_2_SnY (1160.16): C 55.91; H 7.30, N 2.41. Found: C 55.38; H 7.58, N 2.40. ^1^H NMR (400 MHz, THF-*d*_8_): *δ* (ppm) = 6.92–6.67 (m, 10H, C*H*_Ph_), 6.27 (s, 8H, C*H*_COT_), 3.63-3.60 (*thf*), 3.56 (s, 12H, C*H*_2 cryptand_), 3.51 (t, J = 4.6 Hz, 12H, C*H*_2 cryptand_), 2.52 (t, J = 4.8 Hz, 12H, C*H*_2 cryptand_), 0.69 (s, 18H, Si*tBu*Me_2_), 0.39 (s, 6H, Si*t*Bu*Me*_2_), −0.36 (s, 6H, Si*t*Bu*Me*_2_). ^13^C{^1^H} NMR (101 MHz, THF-*d*_8_): *δ* (ppm) = 173.7 (d, *J*_C-_ = 5.4 Hz, *C*_a_), 152.0 (*C*_b_), 151.3 (*C*_Ph_), 125.8 (*C*H_Ph_), 124.0 (*C*H_Ph_), 92.6 (d, *J*_C-_ = 2.1 Hz, *C*H_COT_), 71.5 (*C*H_2 cryptand_), 68.7 (*C*H_2 cryptand_), 55.0 (*C*H_2 cryptand_), 29.8 (SiC(*C*H_3_)_3_Me_2_), 18.2 (Si*C*(CH_3_)_3_Me_2_), 5.7 (Si*t*Bu*Me*_2_), 0.8 (Si*t*Bu*Me*_2_). ^29^Si{^1^H} NMR (80 MHz, C_6_D_6_): *δ* (ppm) = -3.6. ^119^Sn{^1^H} NMR (80 MHz, THF-*d*_8_): *δ* (ppm) = 774.7. ^89^Y NMR (15 MHz, THF-*d*_8_): *δ* (ppm) = –18.6. IR (ATR): $\widetilde{\nu }\,$(cm^−^^1^) = 3043 (w), 3010 (w), 2946 (s), 2918 (s), 2879 (s), 2840 (s), 2759 (w), 2728 (w), 2694 (w), 2323 (w), 2166 (w), 2115 (w), 2050 (w), 1983 (w), 1738 (w), 1595 (m), 1489 (sh), 1473 (m), 1442 (m), 1382 (w), 1354 (s), 1298 (m), 1257 (m), 1236 (s), 1188 (w), 1132 (m), 1101 (vs), 1207 (w), 1106 (w), 983 (w), 950 (s), 933 (m), 896 (w), 821 (s), 800 (s), 761 (m), 697 (s), 655 (w), 626 (w), 589 (w), 568 (w), 523 (w), 414 (w).

### Synthesis of complex 2-Er

To a mixture of complex **1-Er** (36.0 mg, 0.039 mmol) and [2.2.2]-cryptand (22.3 mg, 0.60 mmol) was added ca. 1 mL of benzene, and the suspension was kept at room temperature for 1 h. Red-colored single crystals were formed, and the remaining solution was removed carefully with a syringe. The crystals were washed twice with ca. 2 ml of n-hexane to remove the unreacted [2.2.2]-cryptand and dried under vacuum. Crystalline yield: 34.3 mg (71%). Anal. Calcd for C_54_H_84_KN_2_O_6_Si_2_SnEr (1238.51): C 52.37; H 6.84, N 2.26. Found: C 52.22; H 6.67, N 2.25. IR (ATR): $\widetilde{\nu }\,$(cm-1) = 3074 (w), 3044 (w), 3010 (w), 2945 (m), 2918 (m), 2879 (s), 2839 (s), 2757 (w), 2727 (w), 2694 (w), 2324 (w), 2289 (w), 2166 (w), 2147 (w), 2115 (w), 2084 (w), 2040 (w), 1984 (w), 1949 (w), 1843 (w), 1740 (w), 1595 (m), 1572 (w), 1490 (m), 1473 (m), 1459 (m), 1408 (w), 1382 (w), 1354 (s), 1230 (m), 1188 (s), 1133 (m), 1102 (vs), 1077 (s), 1001 (w), 982 (w), 950 (s), 933 (sh), 895 (w), 821 (w), 800 (m), 761 (m), 697 (m), 654 (w), 626 (w), 590 (w), 556 (w), 521 (w), 507 (w), 490 (w), 449 (w), 414 (w).

## Supplementary information

Supporting Information

Description of Additional Supplementary Files

Supplementary Data 1

Supplementary Data 2

Supplementary Data 3

Supplementary Data 4

## Data Availability

The following data are part of the Supporting Information: NMR spectra see Supplementary Information, Figs. S1–S10; IR spectra see Supplementary Information, Figs. S11–S14; X-ray crystallography see Supplementary Information Tables S1, S2 and Figs. S15–S19, Magnetometry see Supplementary Information, Figs. S20–S28; Ab initio calculation results see Supplementary Information, Tables S3–S8. NMR, IR, EA, and magnetometry data that support the findings of this study are available in Radar4Chem with the identifier: 10.22000/7b3mcax84fjn33nc. Crystallographic data for the structures reported in this paper have been deposited with the Cambridge Crystallographic Data Centre as a supplementary publication no. CCDC 2432508 (**1-Y**) (Supplementary Data 1), 2432509 (**1-Er**) (Supplementary Data 2), 2432510 (**2-Y**) (Supplementary Data 3), 2432511 (**2-Er**) (Supplementary Data 4). Copies of the data can be obtained free of charge on application to CCDC, 12 Union Road, Cambridge CB21EZ, UK (fax: ( + (44)1223-336-033; email: deposit@ccdc.cam.ac.uk).
