# Supplementary material for: Magnetic properties of monomeric and polymeric stannolediide yttrium and erbium complexes
Source: Commun Chem. 2025 Nov 21;8:368. doi: 10.1038/s42004-025-01797-4 (PMC12638953; doi:10.1038/s42004-025-01797-4)
Supplement: Supplementary file 1 — Supporting Information [file 42004_2025_1797_MOESM1_ESM.pdf]

Magnetic properties of monomeric and polymeric stannolediide yttrium and erbium complexes

Xiaofei Sun,[a] Sören Schlittenhardt,[b,c] Tingting Ruan,[b] Masaichi Saito,[d] Mario Ruben*[b,e,f] and Peter W. Roesky*[a,b]

[a] Institute for Inorganic Chemistry, Karlsruhe Institute of Technology (KIT), Engesserstr. 15, 76131 Karlsruhe, Germany.

[b] Institute of Nanotechnology, Karlsruhe Institute of Technology (KIT), Hermann-von-Helmholtz-Platz 1, D-76344 Eggenstein-Leopoldshafen, Germany.

[c] Institute of Physical and Theoretical Chemistry, Goethe University Frankfurt, Max-von-Laue-Str. 7, 60438 Frankfurt am Main, Germany.

[d] Department of Chemistry, Graduate School of Science and Engineering, Saitama University, Shimo-okubo, Sakura-ku, Saitama city, Saitama 338-8570, Japan.

[e] Centre Européen de Science Quantique (CESQ), Institut de Science et d'Ingénierie Supramoléculaires (ISIS, UMR 7006), CNRS-Université de Strasbourg, 8 Allée Gaspard Monge BP 70028, 67083 Strasbourg Cedex, France.

[f] Institute of Quantum Materials and Technologies (IQMT), Karlsruhe Institute of Technology (KIT), Hermann-von-Helmholtz-Platz 1, 76344 Eggenstein-Leopoldshafen, Germany.

Table of Contents

I. NMR spectra S3

II. IR spectra S8

III. X-ray crystallography S10

III.1 General methods S10

III.2 Summary of crystal data S11

III.3 Comparison of the polymeric structure of Er-germole and Er-stannole **1-Er** S16

IV. Magnetometry S17

IV.1 General methods S17

IV.2 Magnetic measurements of **1-Er** S17

IV.3 Magnetic measurements of **2-Er** S20

V. Ab initio calculations S22

V.1 General methods S22

V.2 Results S22

V.3 Input structures S26

VI. References S37

I. NMR spectra

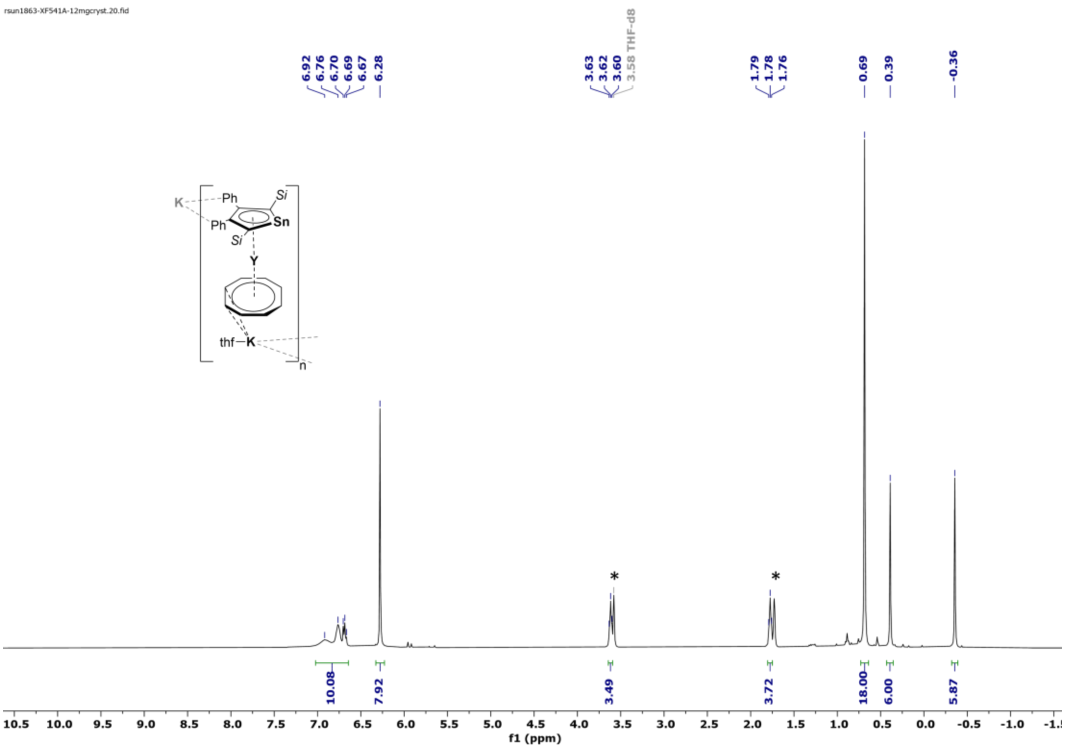

Figure S1.^{1}H NMR spectum of complex **1-Y** in THF-d_8 measured at 298 K. *, residual protio signal of the solvent.

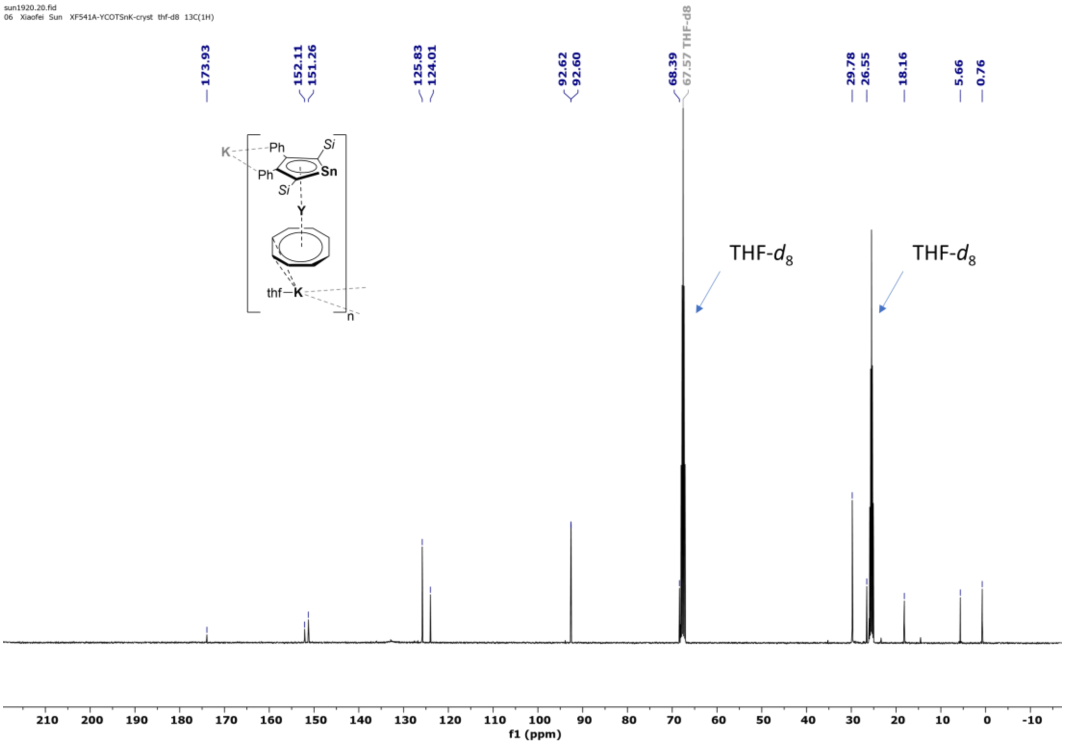

Figure S2. ^{13}C{^{1}H} NMR spectrum of complex **1-Y** in THF-d_8 measured at 298 K.

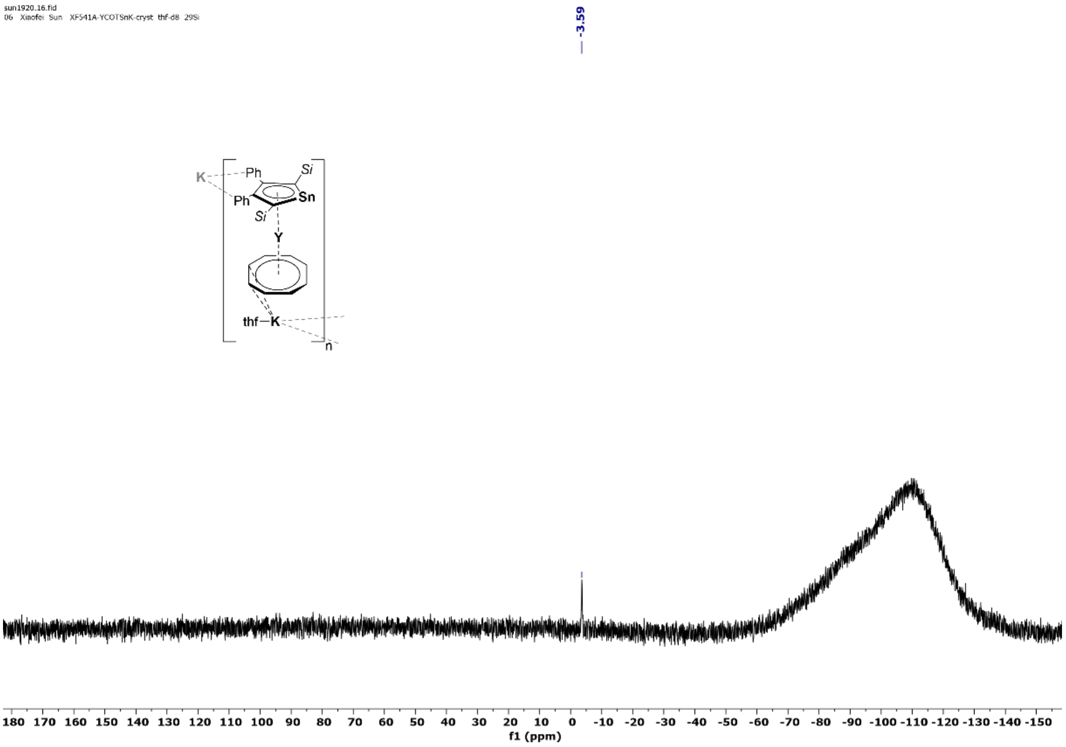

Figure S3. ^{29}Si{^{1}H} NMR spectum of complex **1-Y** in THF-d_8 measured at 298 K.

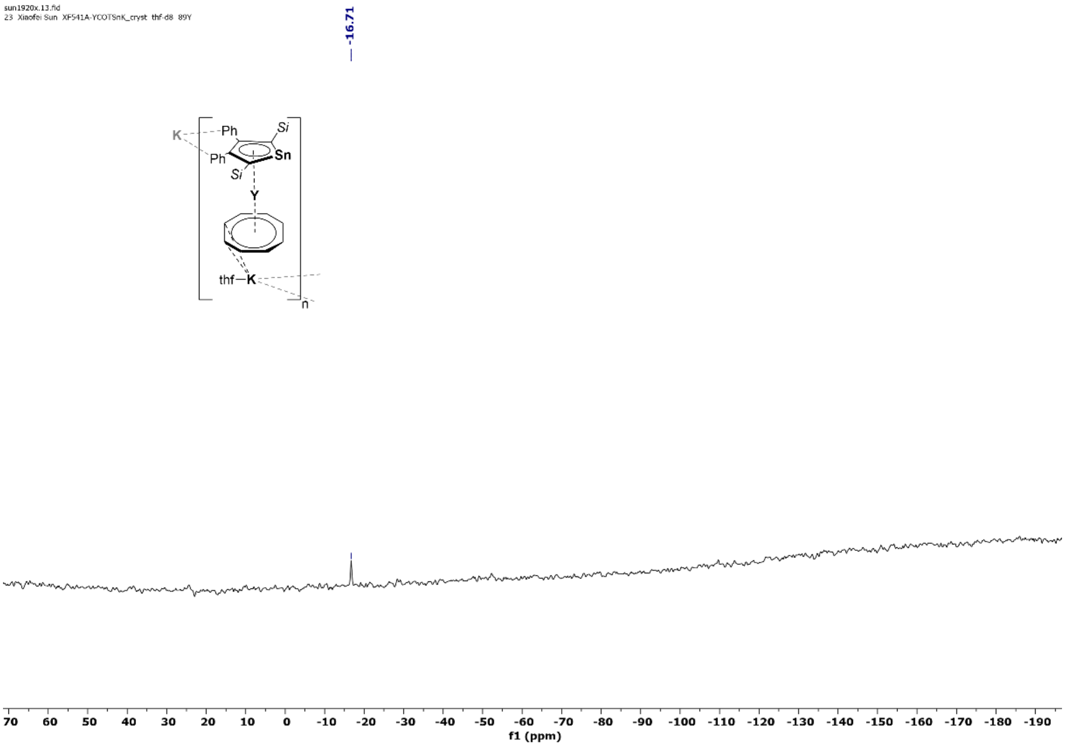

Figure S4. ^{89}Y NMR spectum of complex **1-Y** in THF-d_8 measured at 298 K.

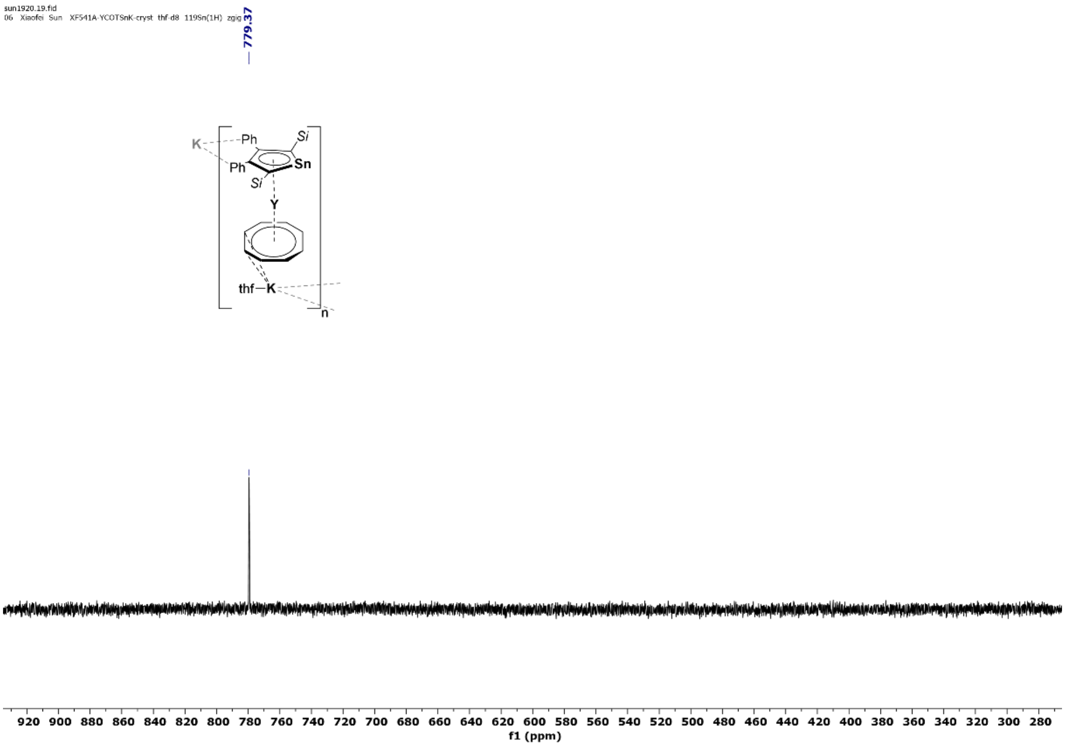

Figure S5. ^{119}Sn{^{1}H} NMR spectum of complex **1-Y** in THF-d_8 measured at 298 K.

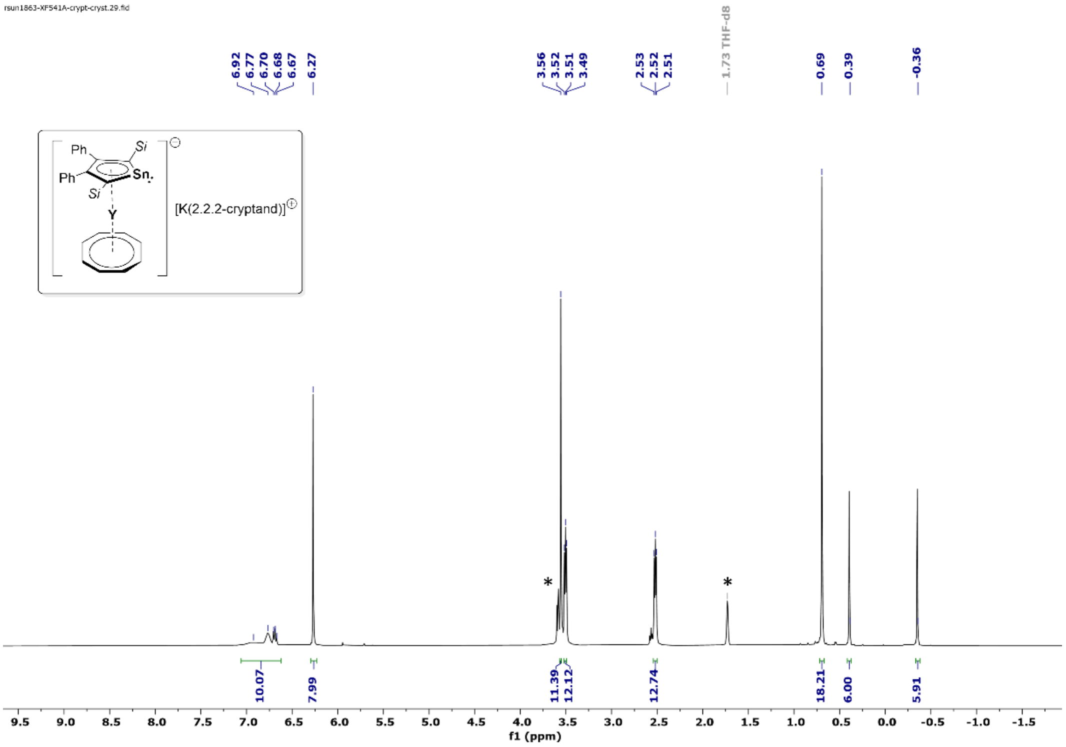

Figure S6. ^{1}H NMR spectum of complex **2-Y** in THF-d_8 measured at 298 K. *, residual protio signal of the solvent.

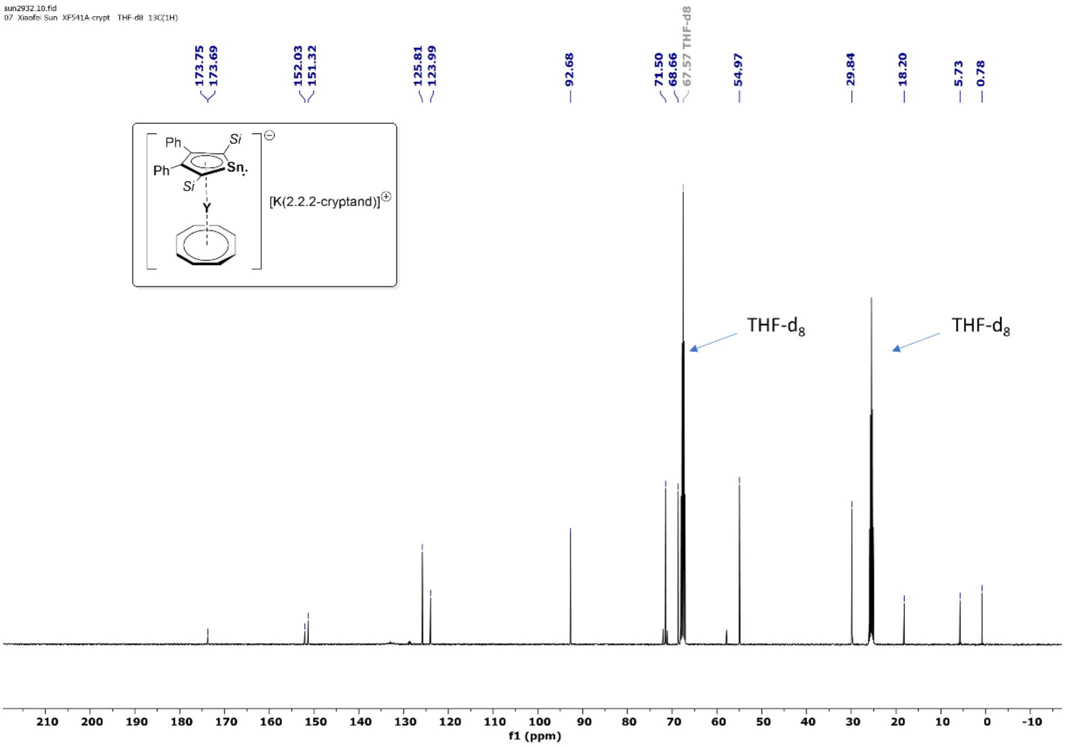

Figure S7. $^{13}C\{^1H\}$ NMR spectrum of complex **2-Y** in THF-d_8 measured at 298 K.

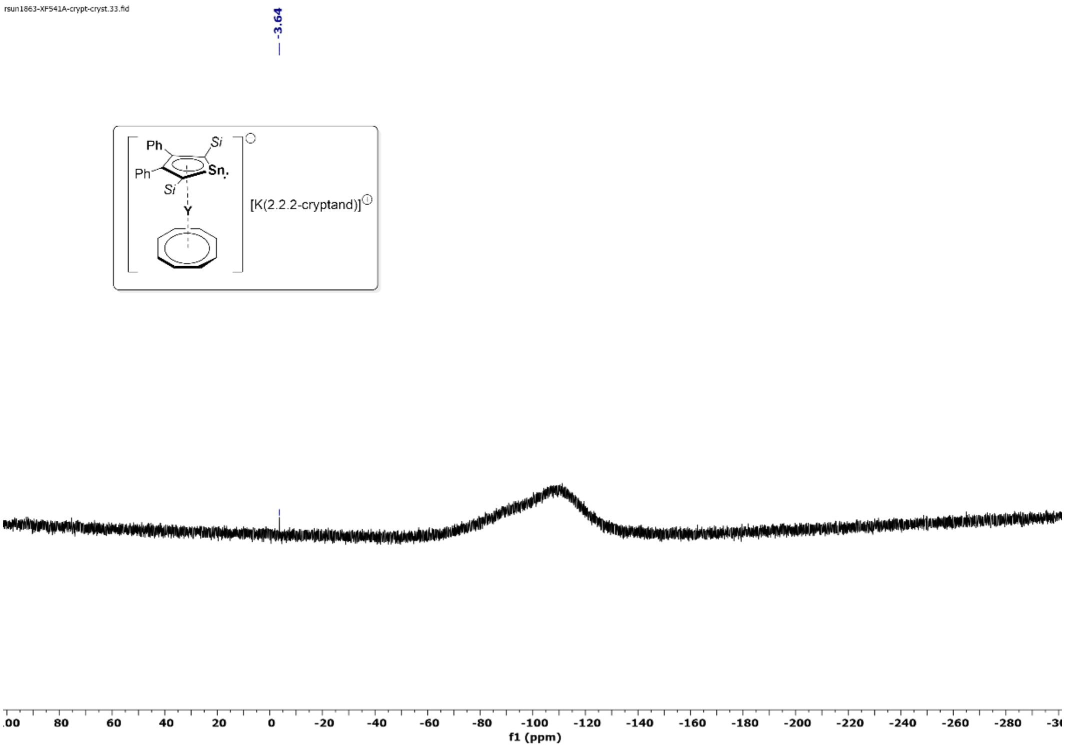

Figure S8. $^{29}Si\{^1H\}$ NMR spectum of complex **2-Y** in THF-d_8 measured at 298 K.

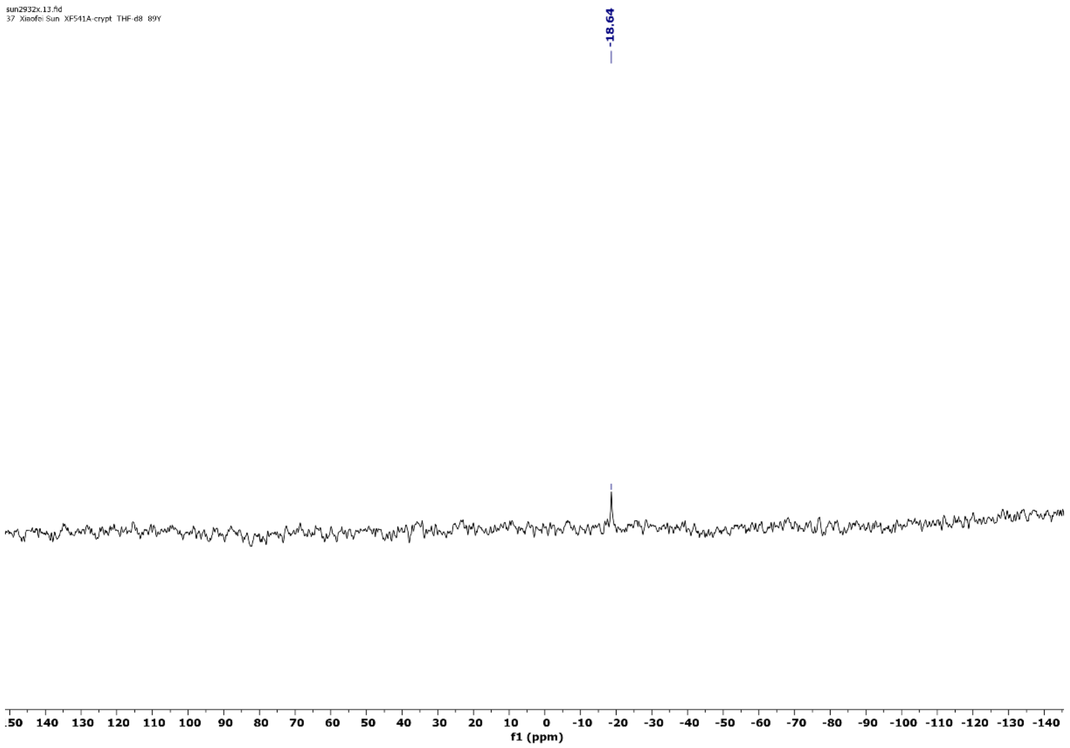

Figure S9. ^{89}Y NMR spectum of complex **2-Y** in THF-d_8 measured at 298 K.

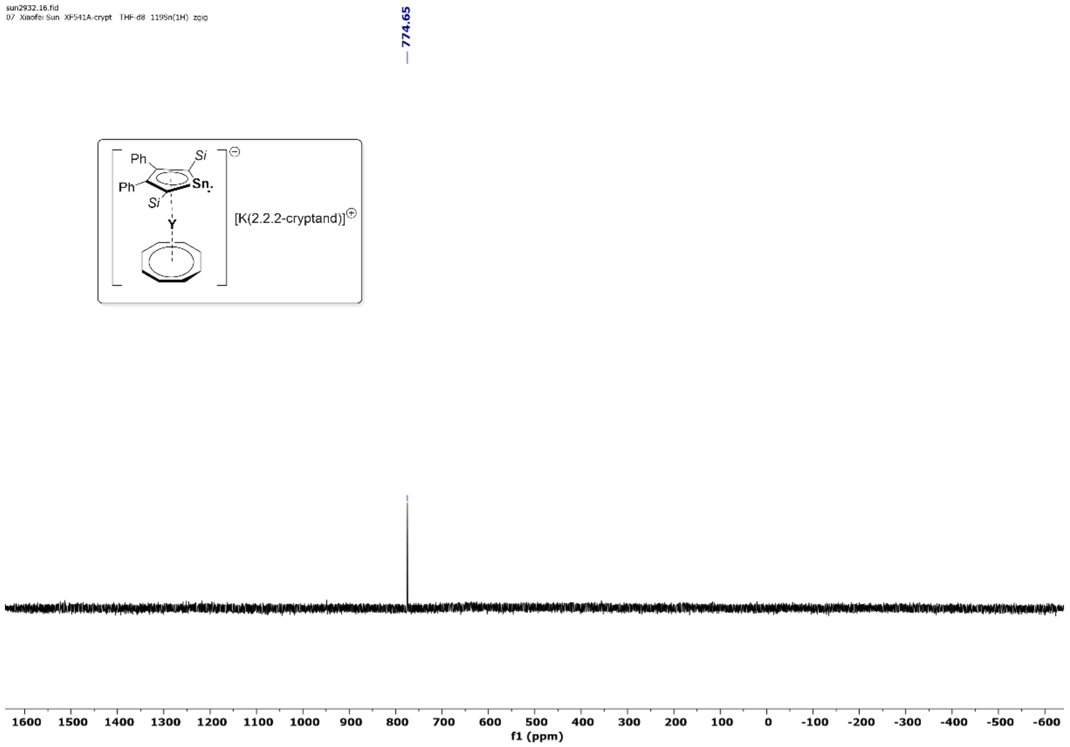

Figure S10. ^{119}Sn{^{1}H} NMR spectum of complex **2-Y** in THF-d_8 measured at 298 K.

II. IR spectra

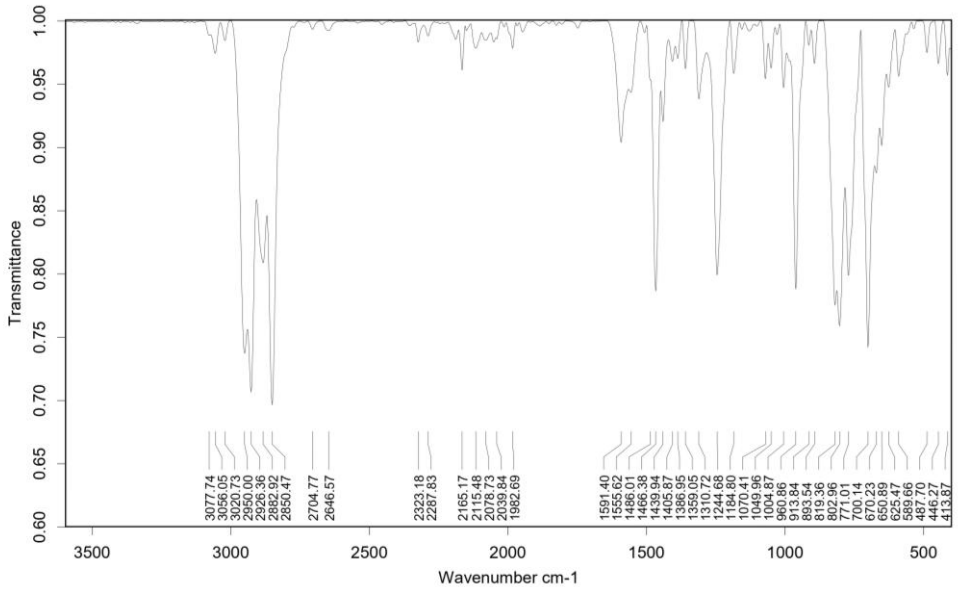

Figure S11. IR spectrum of complex **1-Y**.

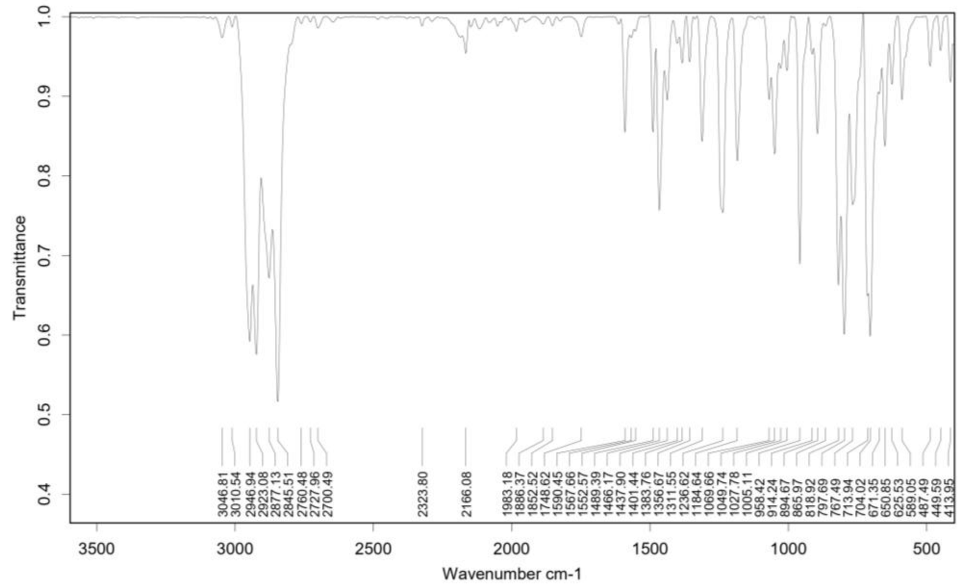

Figure S12. IR spectrum of complex **1-Er**.

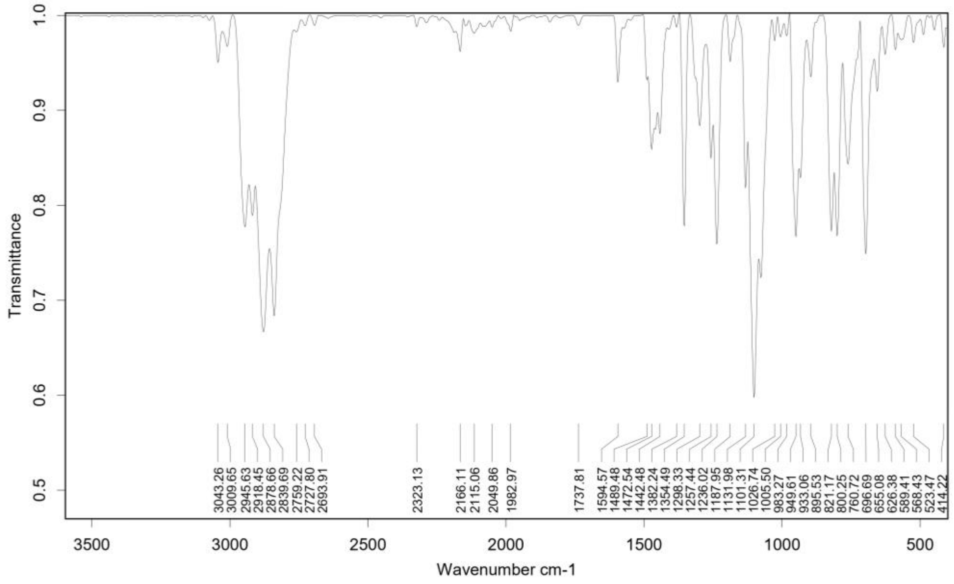

Figure S13. IR spectrum of complex **2-Y**.

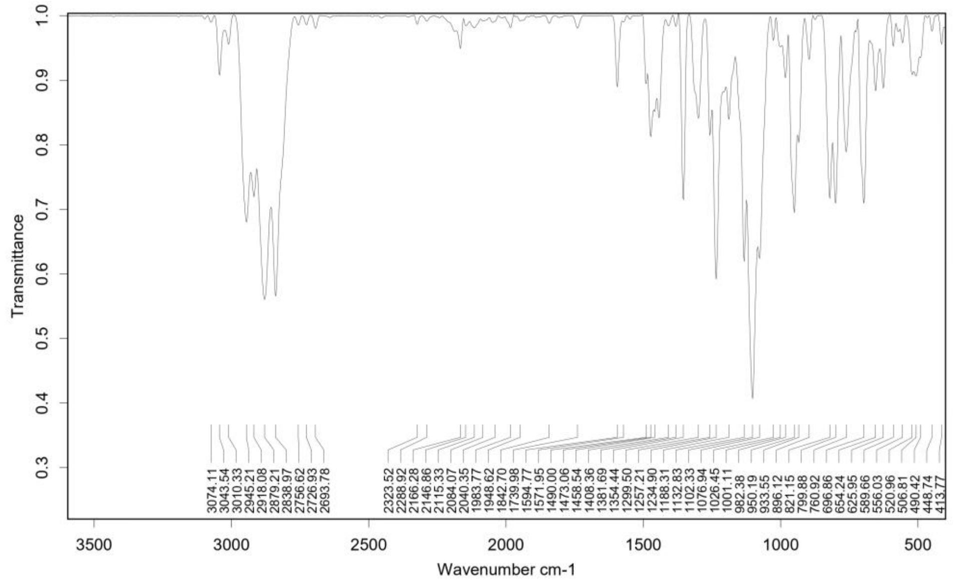

Figure S14. IR spectrum of complex **2-Er**.

III. X-ray crystallography

III.1 General methods

Suitable crystals for the X-ray analysis of all compounds were obtained as described above. A suitable crystal was covered in mineral oil (Aldrich) and mounted on a glass fibre. The crystal was transferred directly to the cold stream of a STOE StadiVari (100 K or 110 K) diffractometer. All structures were solved by using the program SHELXS/T[1, 2] and Olex.[3] The remaining non-hydrogen atoms were located from successive difference Fourier map calculations. The refinements were carried out by using full-matrix least-squares techniques on F_o^2 by using the program SHELXL.[1, 2] The H-atoms were introduced into the geometrically calculated positions (SHELXL procedures) unless otherwise stated and refined riding on the corresponding parent atoms. In each case, the locations of the largest peaks in the final difference Fourier map calculations, as well as the magnitude of the residual electron densities, were of no chemical significance. Specific comments for each data set are given below. Summary of the crystal data, data collection and refinement for compounds are given in Table S1.

Crystallographic data for the structures reported in this paper have been deposited with the Cambridge Crystallographic Data Centre as a supplementary publication no. CCDC 2432508-2432511. Copies of the data can be obtained free of charge on application to CCDC, 12 Union Road, Cambridge CB21EZ, UK (fax: (+(44)1223-336-033; email: deposit@ccdc.cam.ac.uk).

The following special comments were applied to the models of the structures:

In the structure of complex **1-Y**, a half of a THF molecule (O1, C19-C22) is disordered over two positions in a ratio of 0.17/0.33.

In the structure of complex **1-Er**, a half of a THF molecule (O1, C19-C22) is disordered over two positions in a ratio of 0.09/0.41.

In the structure of complex **2-Y**, a half of a C_2H_4 (C53-C54) is disordered over two positions in a ratio of 0.22/0.28.

In the structure of complex **2-Er**, a half of a C_2H_4 (C53-C54) is disordered over two positions in a ratio of 0.20/0.30.

III.2 Summary of crystal data

Table S1. Crystal data, data collection and refinement for compounds **1-M** and **2-M**.

**Compound**	**1-Y**	**1-Er**	**2-Y**	**2-Er**
Formula	$C_{40}H_{56}KOSi_2SnY$	$C_{40}H_{56}ErKOSi_2Sn$	$C_{57}H_{87}KN_2O_6Si_2SnY$	$C_{57}H_{87}ErKN_2O_6Si_2Sn$
CCDC Numer	2432508	2432509	2432510	2432511
$D_{calc.}$/ g cm^{-3}	1.426	1.561	1.346	1.430
μ/mm^{-1}	2.269	2.919	1.556	1.984
Formula Weight	855.72	934.07	1199.16	1277.51
Colour	red	red	orange	orange
Shape	block-shaped	plate-shaped	block-shaped	plate-shaped
Size/mm^3	0.35×0.23×0.10	0.14×0.08×0.03	0.35×0.25×0.24	0.06×0.05×0.03
T/K	115	110	110	110
Crystal System	orthorhombic	orthorhombic	monoclinic	monoclinic
Space Group	*Pnma*	*Pnma*	$P2_1/m$	$P2_1/m$
a/Å	14.2763(4)	14.2635(6)	10.5104(2)	10.5302(5)
b/Å	19.2206(7)	19.2383(11)	17.6611(5)	17.6564(6)
c/Å	14.5229(5)	14.4841(6)	15.9895(4)	16.0171(9)
α/°	90	90	90	90
β/°	90	90	94.326(2)	94.990(4)
γ/°	90	90	90	90
V/Å^3	3985.1(2)	3974.5(3)	2959.60(13)	2966.7(2)
Z	4	4	2	2
Z'	0.5	0.5	0.5	0.5
Wavelength/Å	0.71073	0.71073	0.71073	0.71073
Radiation type	MoK_α	MoK_α	Mo K_α	Mo K_α
Θ_{min}/°	2.000	2.004	1.721	1.941
Θ_{max}/°	25.998	25.996	25.997	26.000
Measured Refl's.	20635	22609	19998	18992
Indep't Refl's	4032	4023	5991	5987
Refl's I≥2 σ(I)	2629	2864	5101	3959
R_{int}	0.0873	0.0708	0.0214	0.0650
Parameters	286	286	660	666
Restraints	186	177	511	579
Largest Peak	0.883	0.614	0.297	0.732
Deepest Hole	-1.008	-0.581	-0.406	-1.274
GooF	1.098	0.950	1.038	1.014
wR_2 (all data)	0.1401	0.0797	0.0520	0.1072
wR_2	0.1110	0.0718	0.0483	0.0884
R_1 (all data)	0.1162	0.0654	0.0364	0.1021
R_1	0.0595	0.0367	0.0248	0.0504

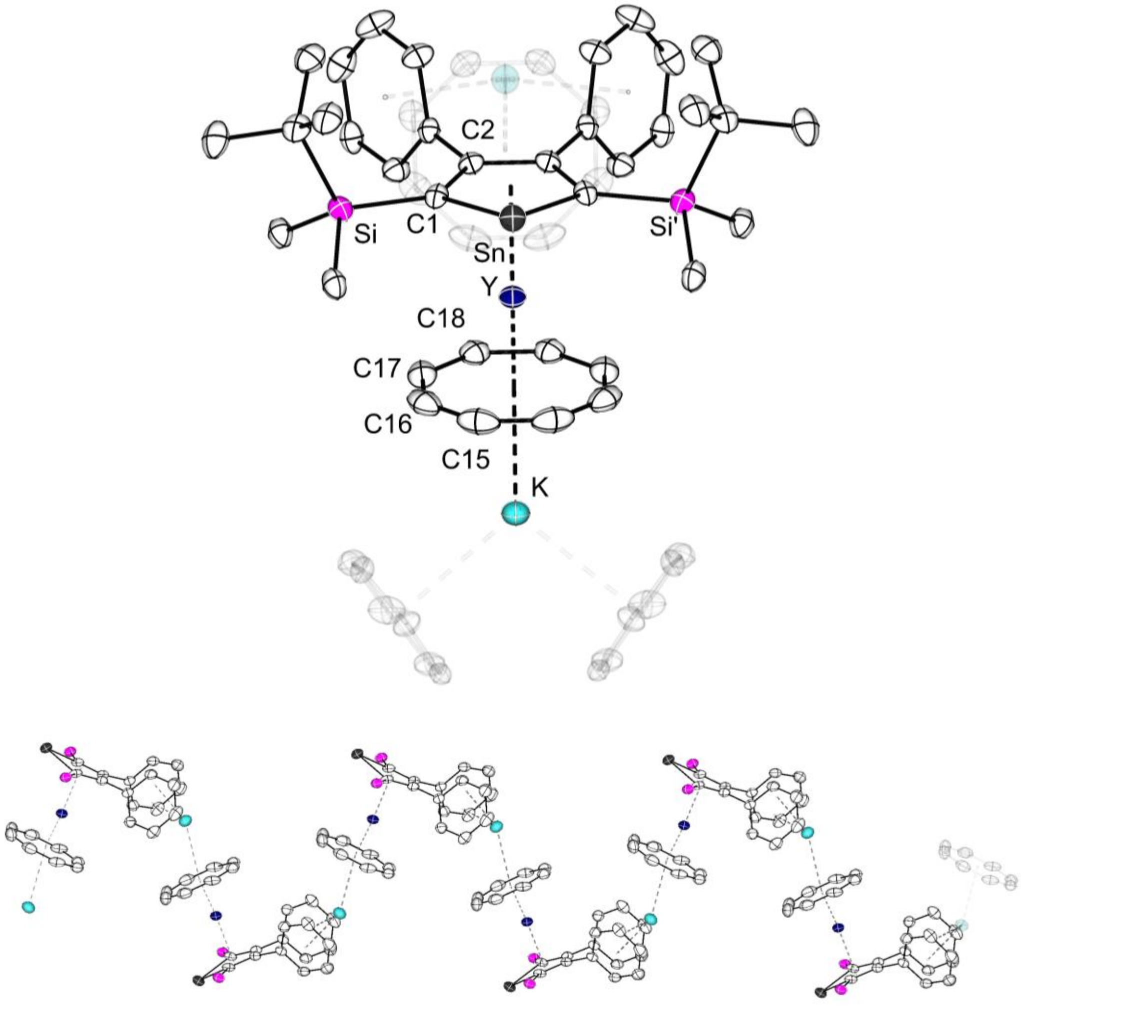

Figure S15. Molecular structure of the complex **1-Y** in the solid state with thermal ellipsoids at 40% level. Asymmetric unit (top) and section of the coordination polymer (bottom). All hydrogen atoms are omitted for clarity. Selected bond distances [Å] and angles [°]: Y–C1 2.629(6), Y–C2 2.609(6), Y–C15 2.564(7), Y–C16 2.570(8), Y–C17 2.594(7), Y–C18 2.590(7), Sn–C1 2.180(7), C1–C2 1.435(9), C2–C2' 1.431(14), C15–C16 1.405(12), C16–C17 1.402(11), C17–C18 1.413(10), K–C17 3.259(8), K–C18 3.085(7); C1–Sn–C1' 79.3(4), Sn–C1–C2 111.2(5), C1–C2–C2' 118.1(4).

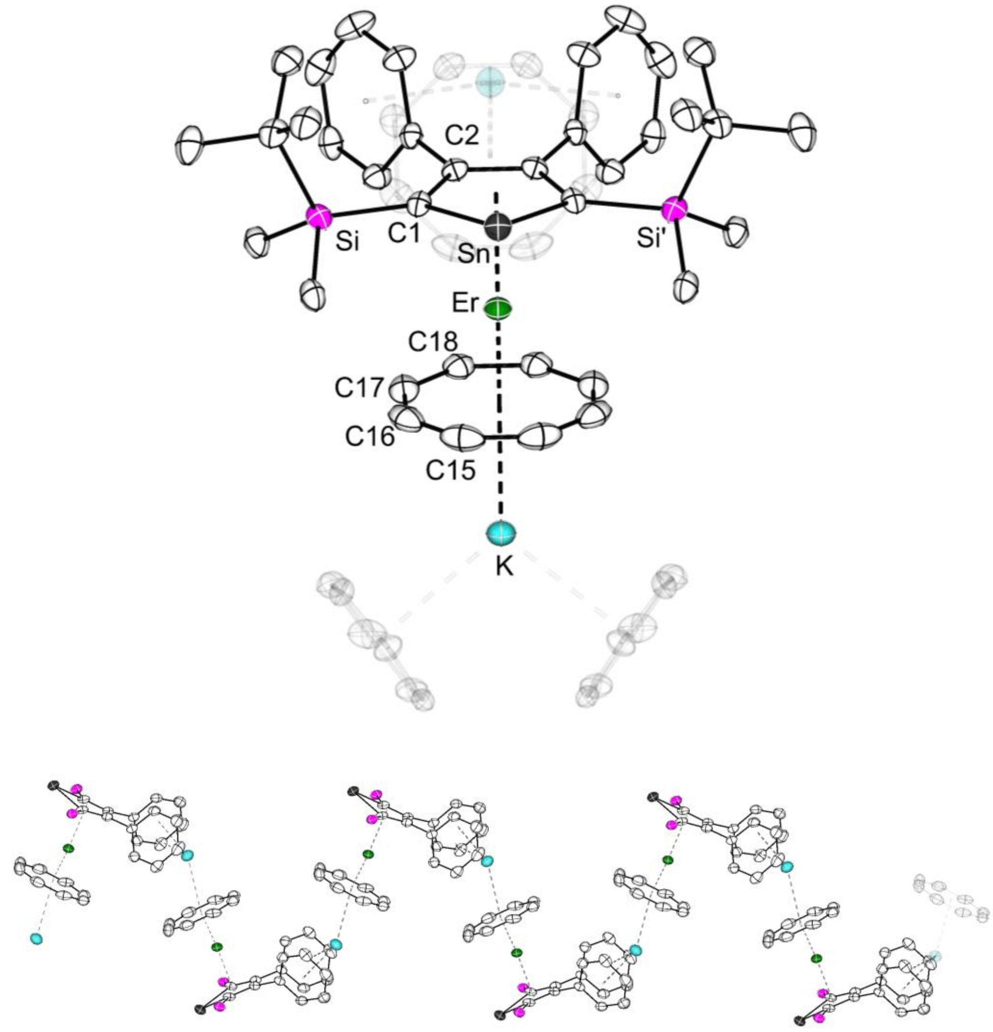

Figure S16. Molecular structure of the complex **1-Er** in the solid state with thermal ellipsoids at 40% level. Only the asymmetric unit of the polymeric structure is depicted here. All hydrogen atoms are omitted for clarity. Selected bond distances [Å] and angles [°]: Er–C1 2.611(5), Er–C2 2.580(4), Er–C15 2.539(5), Er–C16 2.552(5), Er–C17 2.568(5), Er–C18 2.567(5), Sn–C1 2.178(5), C1–C2 1.454(6), C2–C2' 1.408(9), C15–C16 1.392(8), C16–C17 1.396(7), C17–C18 1.385(7), K–C17 3.250(5), K–C18 3.100(5); C1–Sn–C1' 80.0(2), Sn–C1–C2 110.4(3), C1–C2–C2' 118.6(3).

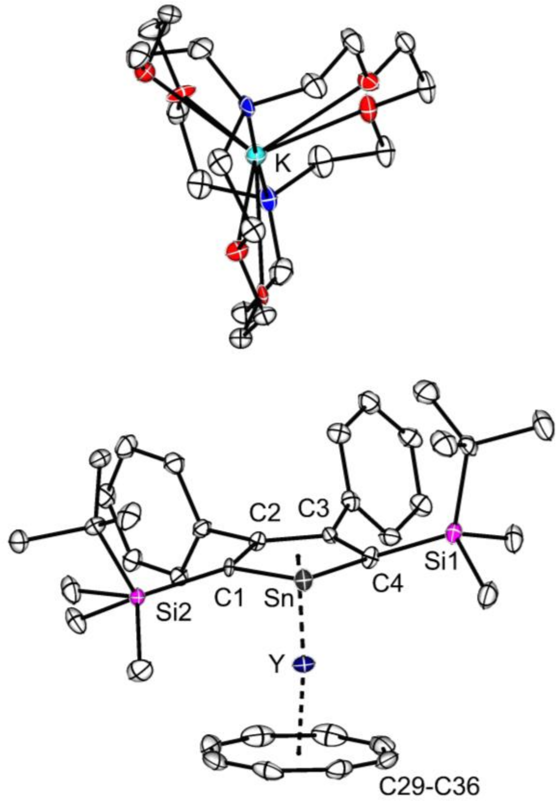

Figure S17. Molecular structure of the complex **2-Y** in the solid state with thermal ellipsoids at 40% level. All hydrogen atoms are omitted for clarity. Selected bond distances [Å] and angles [°]: Y–C1 2.63(2), Y–C2 2.62(2), Y–C3 2.57(2), Y–C4 2.64(2), Y–C29 2.516(6), Y–C30 2.512(5), Y–C31 2.517(6), Y–C32 2.549(11), Y–C33 2.604(10), Y–C34 2.621(6), Y–C35 2.608(5), Y–C36 2.573(6) , Sn–C1 2.12(2), Sn–C4 2.22(2), C1–C2 1.59(2), C2–C3 1.434(6), C3–C4 1.27(2), C29–C30 1.399(5), C30–C31 1.403(5), C31–C32 1.399(5), C32–C33 1.400(5), C33–C34 1.399(5), C34–C35 1.392(5), C35–C36 1.396(5), C29–C36 1.397(5); C1–Sn–C4 79.38(12), Sn–C1–C2 110.4(12), C1–C2–C3 112(2), C2–C3–C4 124(2), C3–C4–Sn 112.8(13).

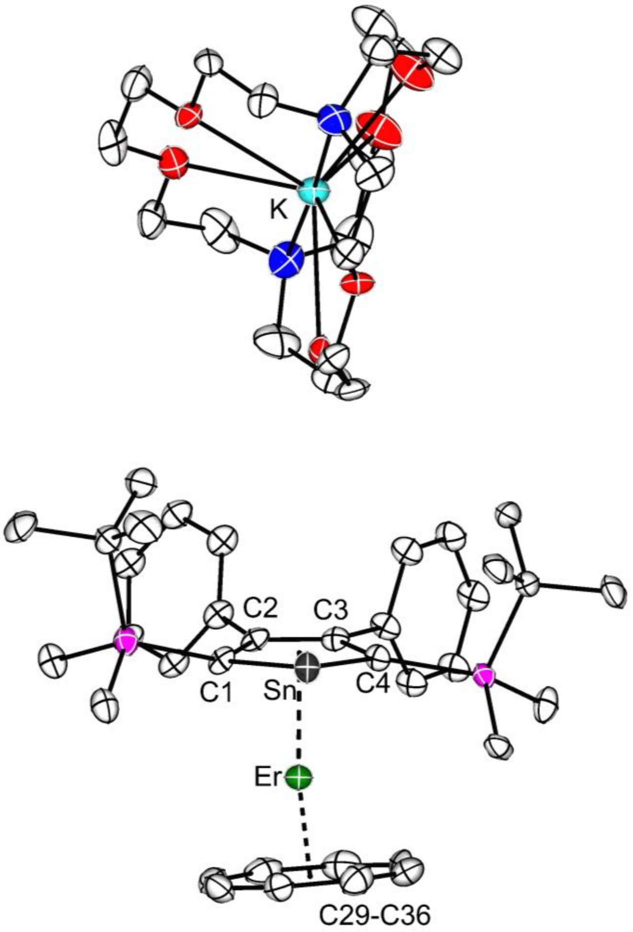

Figure S18. Molecular structure of the complex **2-Er** in the solid state with thermal ellipsoids at 40% level. All hydrogen atoms are omitted for clarity. Selected bond distances [Å] and angles [°]: Er–C1 2.64(3), Er–C2 2.57(4), Er–C3 2.60(4), Er–C4 2.60(3), Er–C29 2.49(2), Er–C30 2.492(15), Er–C31 2.49(2), Er–C32 2.53(2), Er–C33 2.58(2), Er–C34 2.597(15), Er–C35 2.569(15), Er–C36 2.520(18), Sn–C1 2.08(3), Sn–C4 2.64(3), C1–C2 1.45(5), C2–C3 1.425(15), C3–C4 1.43(5), C29–C30 1.392(8), C30–C31 1.391(7), C31–C32 1.393(7), C32–C33 1.385(7), C33–C34 1.392(7), C34–C35 1.389(7), C35–C36 1.387(7), C29–C36 1.387(7); C1–Sn–C4 79.7(4), Sn–C1–C2 113(2), C1–C2–C3 120(4), C2–C3–C4 117(4), C3–C4–Sn 109(2).

III.3 Comparison of the polymeric structure of Er-germole and Er-stannole 1-Er

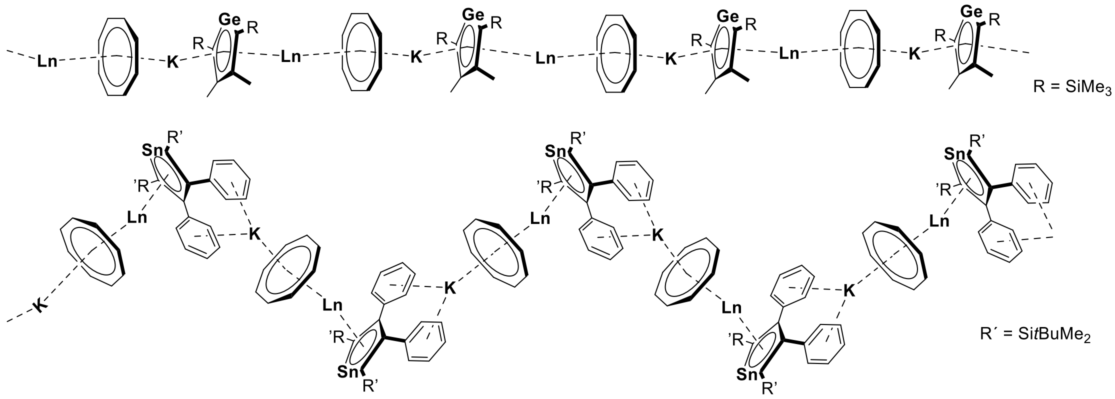

Figure S19. Section of the polymeric structure of the reported Er-germole complex[4] (top) and of the Er-stannole complex **1-Er**.

Table S2. Comparison of the bonding metrics of the reported Er-germole complex[4] (top) and of the Er-stannole complex **1-Er**.

	Er-germole polymer	Er-stannole polymer (**1-Er**)
Er-E (E = Ge or Sn)	2.8863(5) Å	3.1082 Å
Er-C^{COT}	2.507(5)–2.564(5) Å	2.539(5)–2.568(5) Å
K-E (E = Ge or Sn)	3.2979(9) Å	-
K-C^{COT}	3.067(4)–3.351(4) Å	3.100(5)–3.6418(1) Å
$Ct^{metallole}$-Er<br>Ct^{COT}-Er	2.248 Å<br>1.742 Å	2.274 Å<br>1.789 Å
$Ct^{metallole}$-K<br>Ct^{COT}-K	2.857 Å<br>2.632 Å	-<br>2.835 Å

IV. Magnetometry

IV.1 General methods

Temperature dependent as well as field dependent DC magnetic measurements were performed on a Quantum Design MPMS3 SQUID magnetometer, while frequency dependent AC magnetometry was performed on a Quantum Design MPMS XL SQUID magnetometer. All measurements were performed on polycrystalline samples prepared in a glass tube along small amounts of eicosane in a glovebox and subsequently flame sealed using standard Schlenk techniques. The eicosane was gently melted in order to prevent sample movement under the application of a magnetic field.

T-dependence of the susceptibility was measured in a range from 300 K to 2 K upon cooling in an external field of 1000 Oe. The obtained data were corrected for the samples' intrinsic diamagnetism using Pascal constants as well as for the contribution of the sample holder. Field dependent magnetization measurements were performed between 0 T to 7 T using the "stable at each field" function and from 3 T to -3 T back to 3 T using "continuous sweep" at both 20 Oe/s and 100 Oe/s.

AC magnetic measurements were performed with an alternating field of 3.5 Oe without an additional external DC field.

IV.2 Magnetic measurements of 1-Er

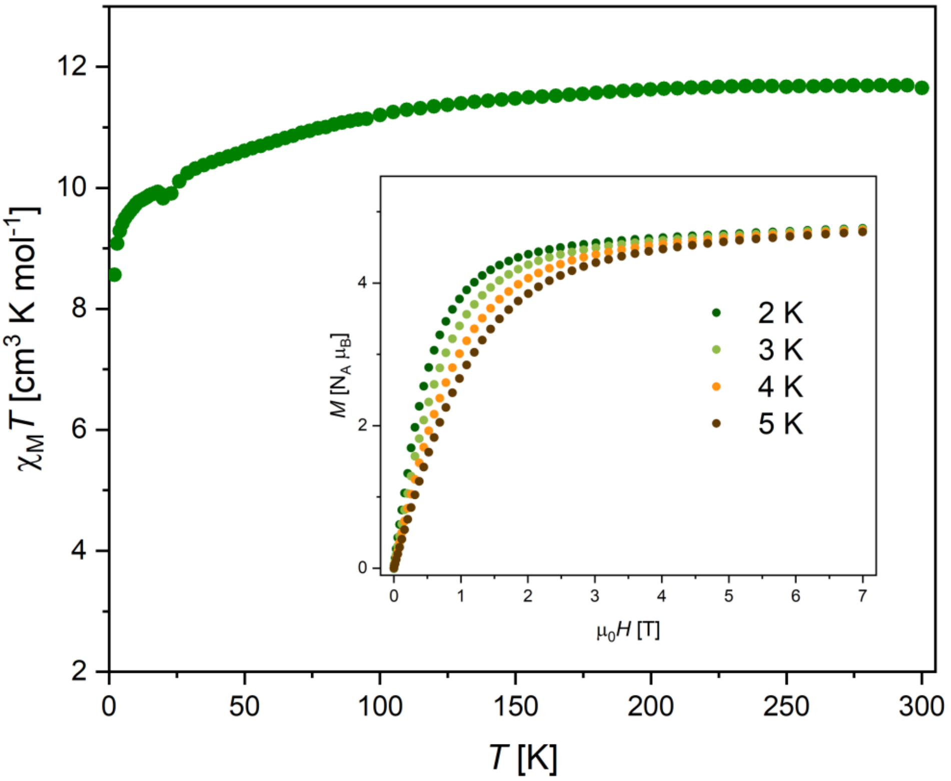

Figure S20. Temperature dependence of the magnetic susceptibility and field-dependent magnetization (inset) for **1-Er**.

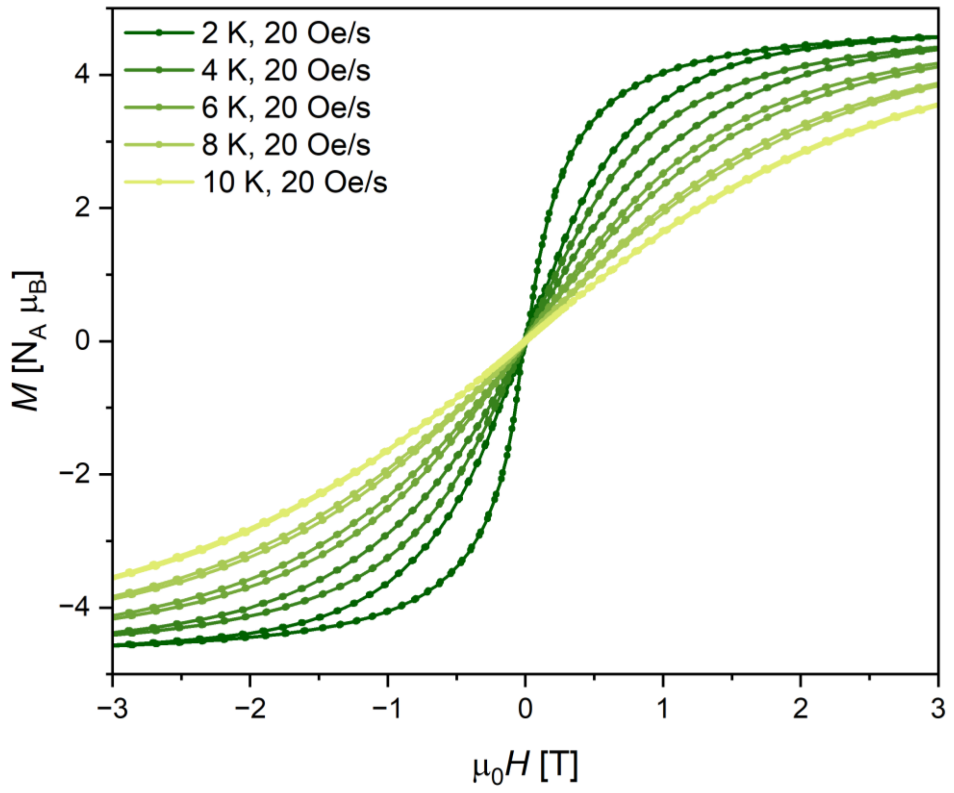

Figure S21. Hysteresis loops recorded using 20 Oe/s for **1-Er**.

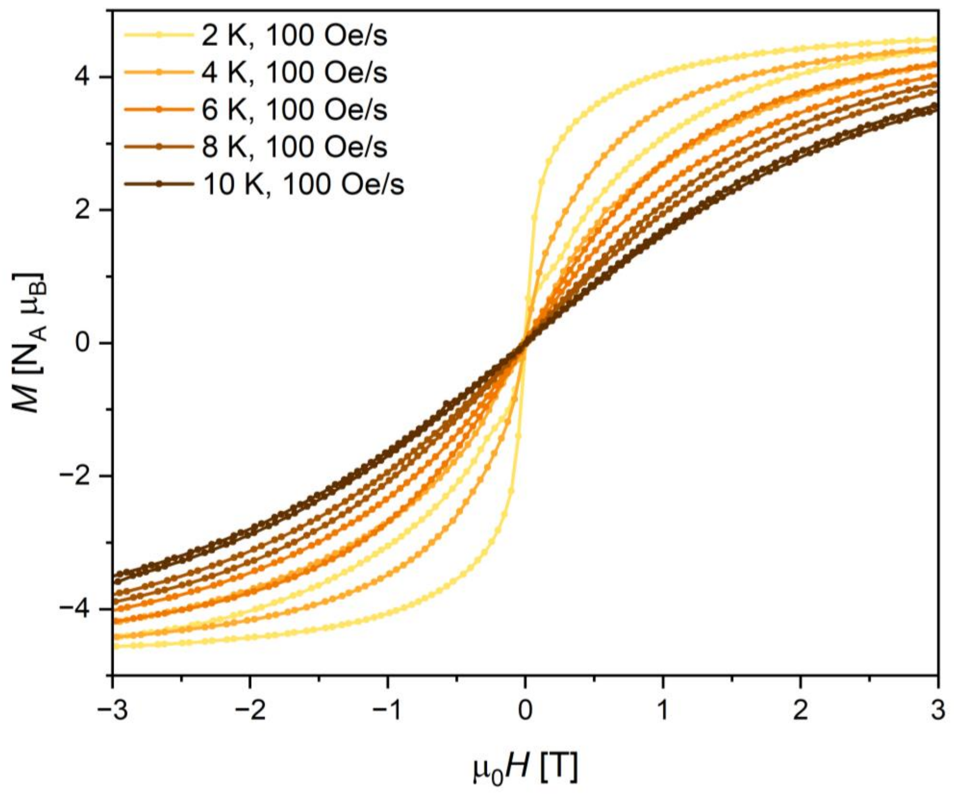

Figure S22. Hysteresis loops recorded using 100 Oe/s for **1-Er**.

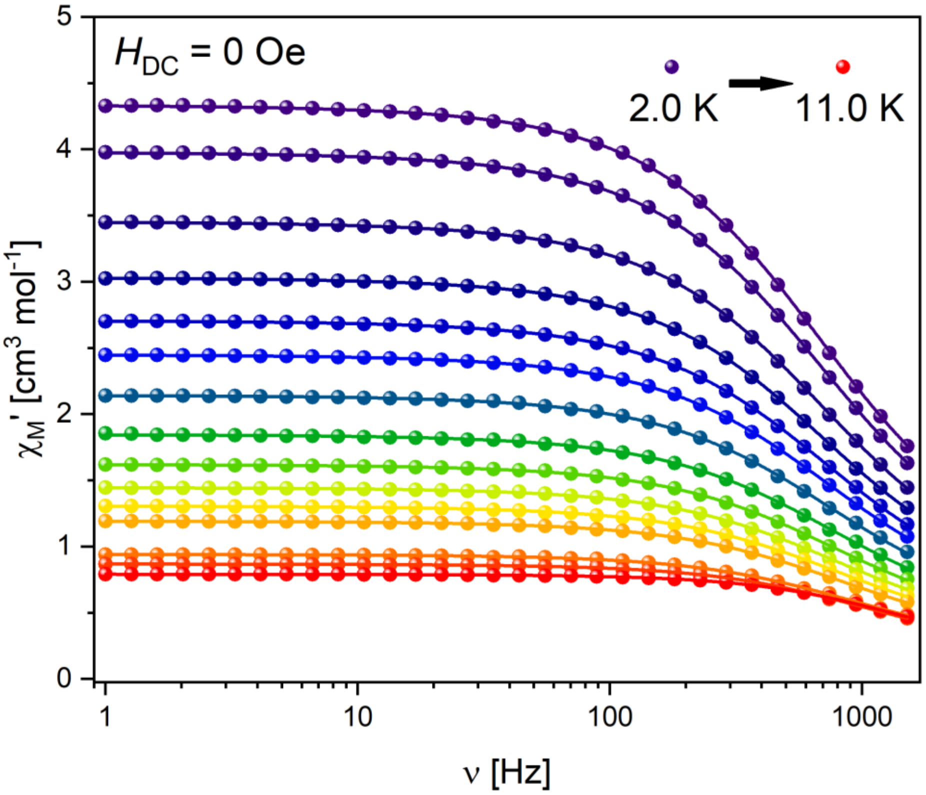

Figure S23. Frequency dependent in-phase component of the magnetic susceptibility for **1-Er**. The solid lines are the best fits to a generalized Debye model.

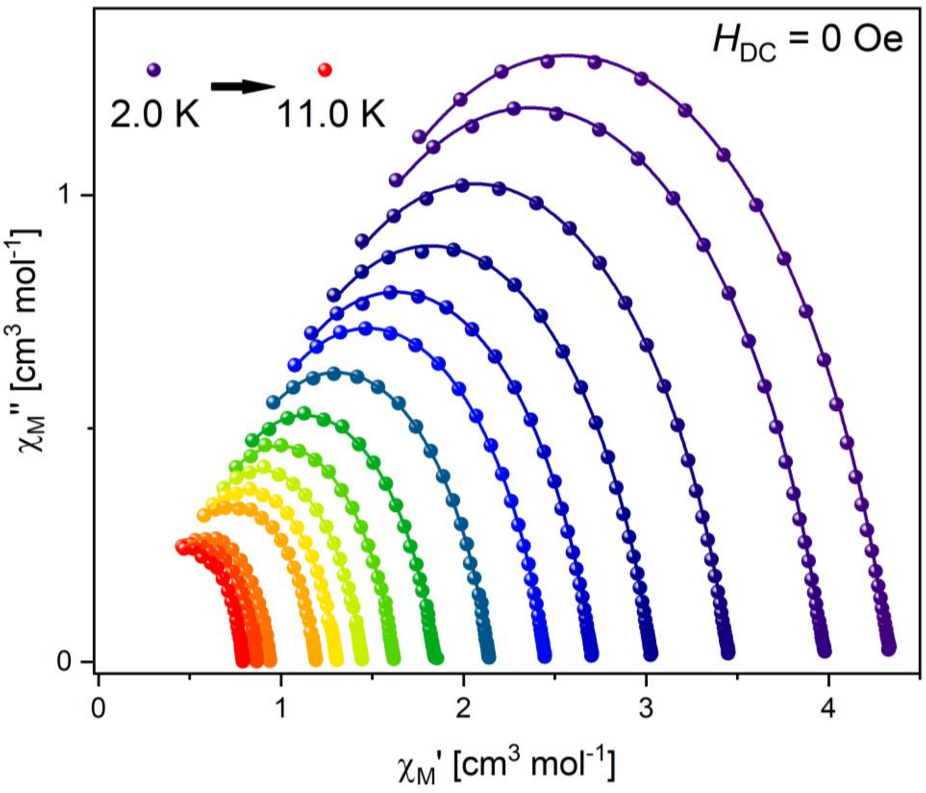

Figure S24. Cole-cole plot for **1-Er**. The solid lines are the best fits to a generalized Debye model.

IV.3 Magnetic measurements of 2-Er

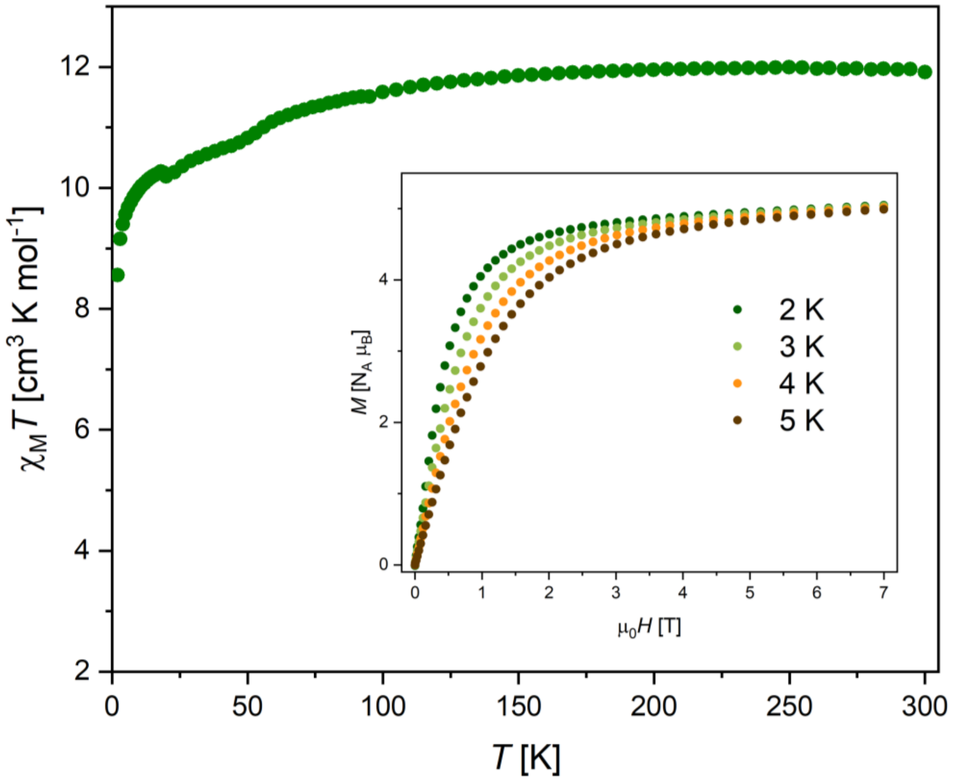

Figure S25. Temperature dependence of the magnetic susceptibility and field-dependent magnetization (inset) for **2-Er**.

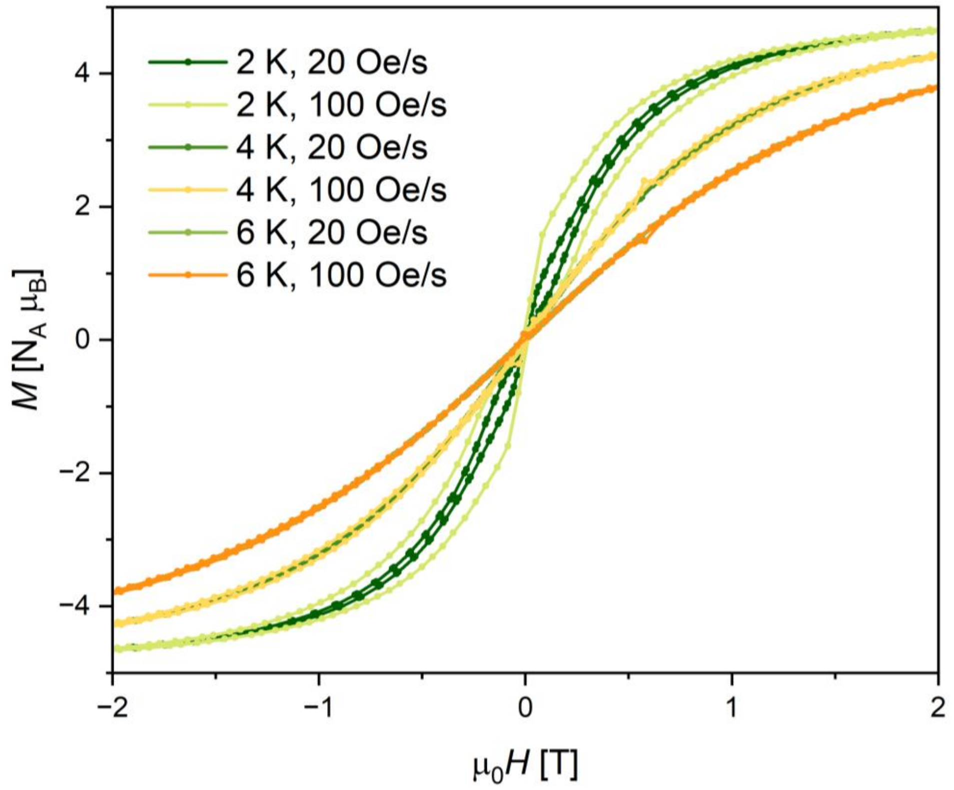

Figure S26. Hysteresis loops recorded for **2-Er**.

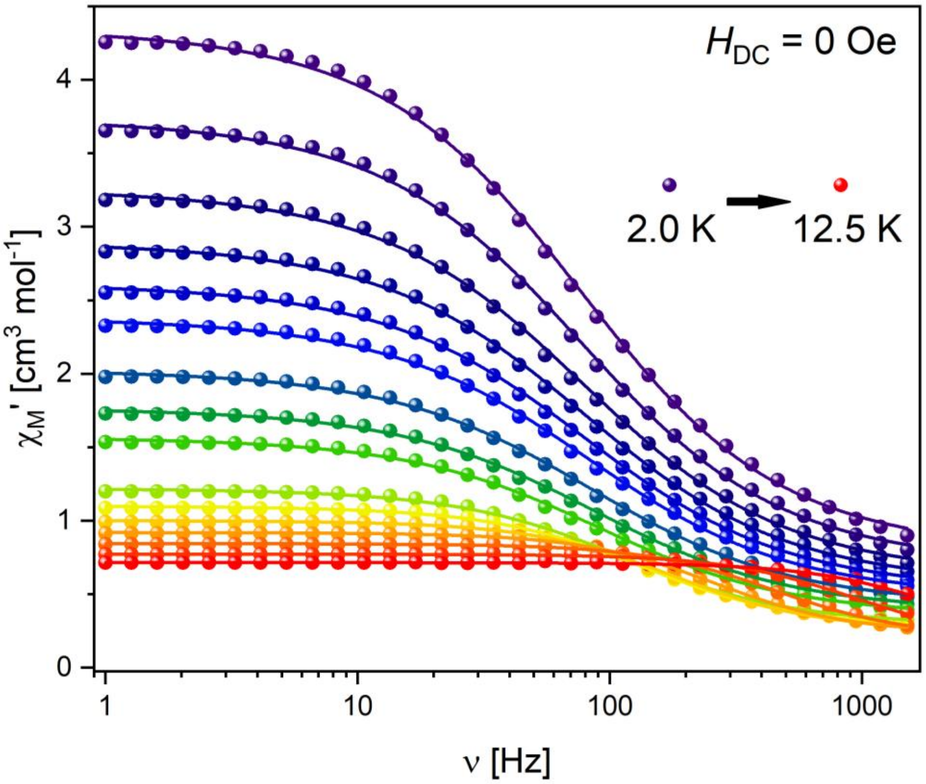

Figure S27. Frequency dependent in-phase component of the magnetic susceptibility for **2-Er**. The solid lines are the best fits to a generalized Debye model.

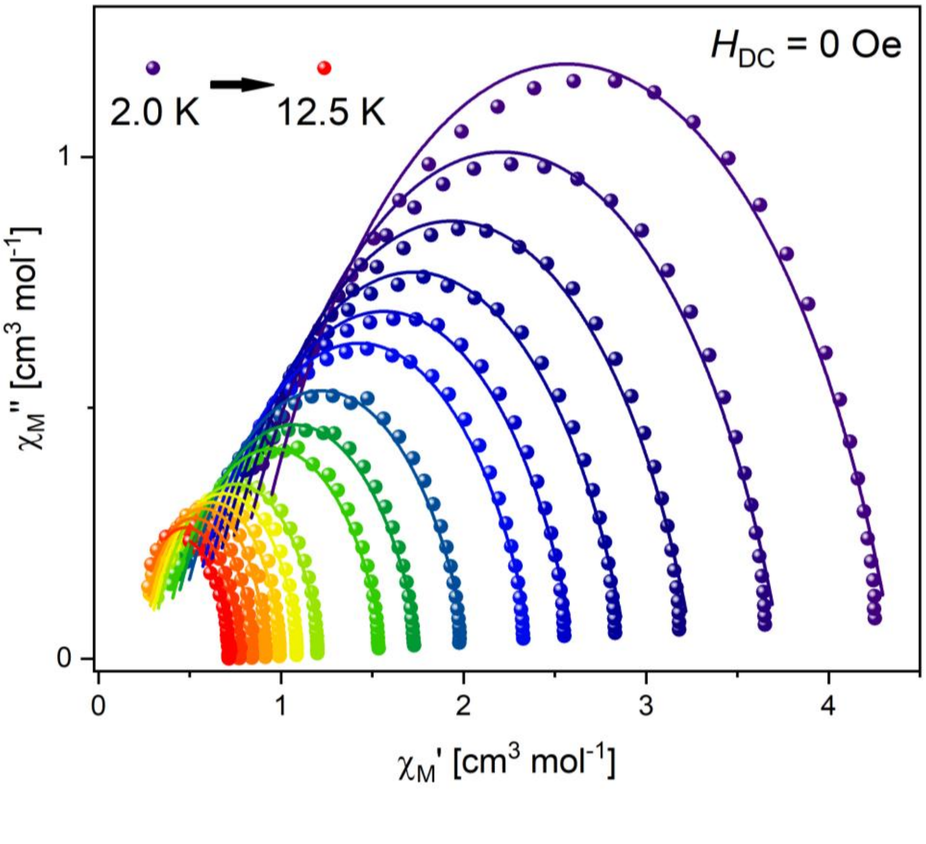

Figure S28. Cole-cole plot for **2-Er**. The solid lines are the best fits to a generalized Debye model.

V. Ab initio calculations

V.1 General methods

All calculations were [11,7]-CASSCF calculations following the CASSCF/RASSI/SINGLE_ANISO routine using OpenMolcas.[5] The input structures were taken as obtained from x-ray crystallography, exchanging substituents as described in the main text and used without geometry optimization. The employed xyz data is provided below. All employed basis sets were of the ANO-RCC type with the following sizes: VTZP (for Er), VDZP (for coordinating C's and Sn) and VDZ (for all others). The CASSCF optimization was performed with the maximum number of available CI roots for each spin state (35 quartets and 112 doublets) of which all were included in the RASSI subroutine.

V.2 Results

Table S3. Energies and g-tensors for **1-Er**.

Doublet state	E [cm^{-1}]	g_x	g_y	g_z
1	0.000	0.0052	0.0065	17.7766
2	92.254	0.4881	1.7175	16.0466
3	98.037	0.0531	2.8515	12.4097
4	115.392	4.1079	5.0763	8.8370
5	138.826	0.0616	0.9521	8.0433
6	170.755	0.0442	0.8215	11.4818
7	198.789	0.0002	0.8328	13.9085
8	214.229	0.3025	1.2348	13.0565

Table S4. Energies and g-tensors for **2-Er**.

Doublet state	E [cm^{-1}]	g_x	g_y	g_z
1	0.000	0.0157	0.0299	17.5795
2	72.273	0.3279	0.4944	15.8834
3	112.999	3.7653	4.0965	10.9310
4	130.307	1.1257	3.7807	10.2386
5	156.909	6.9824	4.9607	2.5755
6	173.630	8.9828	6.0478	1.5421
7	192.393	0.6267	1.3869	13.6003
8	252.172	0.0364	0.0890	16.8074

Table S5. Energies and g-tensors for **2-Er^{TMS}**.

Doublet state	E [cm^{-1}]	g_x	g_y	g_z
1	0.000	0.0155	0.0296	17.5859
2	71.579	0.3149	0.4737	15.8879
3	112.538	3.8131	4.1066	10.8704
4	129.428	1.2029	3.7384	10.2980
5	156.005	2.7350	4.7303	6.9755
6	172.527	8.8379	6.2373	1.5842
7	190.844	0.5621	1.3825	13.4198
8	250.940	0.0372	0.0900	16.7925

Table S6. Energies and g-tensors for **2-Er^{tBu}**.

Doublet state	E [cm^{-1}]	g_x	g_y	g_z
1	0.000	0.0154	0.0270	17.6430
2	66.469	0.2268	0.5585	15.8081
3	106.483	3.8864	5.0458	8.9339
4	119.781	0.2161	3.7318	12.6331
5	140.836	0.9220	2.8539	11.9094
6	155.998	1.8969	3.5070	9.0485
7	186.336	0.3662	0.7606	13.2551
8	249.038	0. 0484	0.1009	16.6033

Table S7. Energies and g-tensors for **2-Er^{Me}**.

Doublet state	E [cm^{-1}]	g_x	g_y	g_z
1	0.000	0.0142	0.0249	17.6751
2	71.820	0.3120	0.6681	15.9685
3	111.705	3.9459	4.9231	9.4305
4	122.911	1.1706	3.7101	11.4184
5	146.546	0.4538	3.4782	11.3118
6	158.976	1.7040	3.9495	8.5059
7	189.567	0.3875	0.7926	13.3532
8	252.077	0.0442	0.0958	16.6095

Table S8 Crystal field parameters from *ab initio* CASSCF calculations.

k	q	B_k^q				
		**1-Er**	**2-Er**	**2-Er^{TMS}**	**2-Er^{tBu}**	**2-Er^{Me}**
2	-2	0.22544	-0.08251	-0.07338	0.11963	0.0945
2	-1	0.09063	-0.39132	-0.38881	-0.4904	-0.57626
2	0	-0.1466	-0.30669	-0.30299	-0.1874	-0.22488
2	1	-0.14531	0.69597	0.70233	0.82167	0.78682
2	2	-0.19598	0.67023	0.66344	0.44379	0.51136
4	-4	0.00194	0.00107	0.00119	0.00186	0.00203
4	-3	0.00357	-0.00488	-0.00464	-0.00278	-0.00267
4	-2	0.00259	-0.00146	-0.0014	-7.24352E-4	-8.19525E-4
4	-1	-8.26726E-4	-0.00969	-0.00978	-0.00829	-0.00742
4	0	-0.00544	-0.0048	-0.00478	-0.00505	-0.00506
4	1	0.002	-1.11041E-4	3.87197E-5	0.00151	0.00189
4	2	-0.00241	0.00469	0.00467	0.00444	0.00421
4	3	-0.00181	-0.00885	-0.00829	-0.0041	-0.00389
4	4	2.4701E-4	-0.00207	-0.00207	-0.00243	-0.00223
6	-6	-2.22698E-5	5.93068E-6	6.45828E-6	1.59338E-5	1.23875E-5
6	-5	2.79846E-4	-2.34944E-5	-1.77088E-5	7.07116E-5	5.24889E-5
6	-4	7.95003E-5	3.2059E-5	3.47274E-5	5.16182E-5	5.95418E-5
6	-3	9.39637E-5	-8.97005E-5	-8.75194E-5	-6.99084E-5	-5.6269E-5
6	-2	3.06771E-5	6.03519E-5	5.68275E-5	3.66981E-5	3.28888E-5
6	-1	-1.88909E-5	1.82835E-4	1.82235E-4	1.74487E-4	1.80047E-4
6	0	-2.25614E-5	-2.12362E-5	-2.13582E-5	-2.29957E-5	-2.27407E-5
6	1	3.67237E-5	-1.27337E-4	-1.28483E-4	-1.43163E-4	-1.39642E-4
6	2	-2.50683E-5	-1.55699E-5	-1.36994E-5	1.60831E-5	2.55678E-5
6	3	-4.3131E-5	-1.81162E-4	-1.69994E-4	-8.29724E-5	-7.11498E-5
6	4	1.06071E-5	-7.17132E-5	-7.1184E-5	-8.11292E-5	-7.60033E-5
6	5	1.47939E-4	2.97522E-4	3.02563E-4	3.09473E-4	2.95632E-4
6	6	-2.88191E-5	1.87685E-5	1.86136E-5	1.28807E-5	1.51336E-5

V.3 Input structures

V.3.1 Input structure for **1-Er** in Angstrom.

Er1 0.00 0.00 0.00

Sn1 -0.7732 0.00 3.0105

Sn2 -7.9049 0.00 -7.6104

Sn3 6.3586 0.00 -7.6104

Y1 -7.1317 0.00 -4.5999

Y2 7.1318 0.00 -4.5999

O1 0.7002 0.0808 -5.9745

O2 7.832 -0.0808 1.3747

K1 5.6597 0.00 -0.2352

K2 -1.4721 0.00 -4.3646

C1 0.6318 1.4005 2.1105

C2 1.8313 0.7041 1.676

C3 -2.2737 0.706 -0.8834

C4 -1.3851 1.6814 -1.328

C5 -0.127 1.6853 -1.9335

C6 0.7687 0.7118 -2.3434

C7 0.6318 -1.4006 2.1105

C8 1.8313 -0.7041 1.676

C9 -2.2737 -0.7061 -0.8834

C10 -1.3851 -1.6815 -1.328

C11 -0.127 -1.6853 -1.9335

C12 0.7687 -0.7118 -2.3434

C13 -6.5 1.4005 -6.7103

C14 -5.3004 0.7041 -6.2758

C15 -4.0809 1.4775 -5.8891

C16 -3.0468 1.6602 -6.803

C17 -1.9485 2.4394 -6.5032

C18 -1.8401 3.0743 -5.2779

C19 -2.8214 2.903 -4.3683

C20 -3.9325 2.1123 -4.671

C21 4.858 0.706 -3.7165

C22 5.7467 1.6814 -3.2718

C23 7.0047 1.6853 -2.6664

C24 7.9005 0.7118 -2.2565

C25 0.3053 2.8843 2.2418

C26 3.0509 1.4775 1.2892

C27 4.085 1.6602 2.2032

C28 5.1833 2.4394 1.9034

C29 5.2917 3.0743 0.678

C30 4.3103 2.903 -0.2316

C31 3.1992 2.1123 0.0711

C32 0.3053 -2.8844 2.2418

C33 3.0509 -1.4775 1.2892
C34 4.085 -1.6603 2.2032
C35 5.1833 -2.4394 1.9034
C36 5.2917 -3.0743 0.678
C37 4.3103 -2.9031 -0.2316
C38 3.1992 -2.1124 0.0711
C39 -6.5 -1.4006 -6.7103
C40 -5.3004 -0.7041 -6.2758
C41 -4.0809 -1.4775 -5.8891
C42 -3.0468 -1.6603 -6.803
C43 -1.9485 -2.4394 -6.5032
C44 -1.8401 -3.0743 -5.2779
C45 -2.8214 -2.9031 -4.3683
C46 -3.9325 -2.1124 -4.671
C47 4.858 -0.7061 -3.7165
C48 5.7467 -1.6815 -3.2718
C49 7.0047 -1.6853 -2.6664
C50 7.9005 -0.7118 -2.2565
C51 1.5774 1.06 -6.5742
C52 2.0253 0.2885 -7.8792
C53 2.322 -1.0889 -7.2448
C54 1.5061 -1.0928 -5.9224
C55 8.7092 -1.0601 1.9743
C56 9.1571 -0.2886 3.2794
C57 9.4537 1.0889 2.6449
C58 8.6379 1.0927 1.3225
C59 -9.4055 0.706 -3.7165
C60 -8.5168 1.6814 -3.2718
C61 -7.2588 1.6853 -2.6664
C62 -6.363 0.7118 -2.2565
C63 7.7635 1.4005 -6.7103
C64 8.9631 0.7041 -6.2758
C65 -9.4055 -0.7061 -3.7165
C66 -8.5168 -1.6815 -3.2718
C67 -7.2588 -1.6853 -2.6664
C68 -6.363 -0.7118 -2.2565
C69 7.7635 -1.4006 -6.7103
C70 8.9631 -0.7041 -6.2758
H1 -6.7034 2.3249 -6.7921
H2 -3.0983 1.2385 -7.6529
H3 -1.2576 2.5418 -7.1467
H4 -1.0883 3.6223 -5.0813
H5 -2.7556 3.3197 -3.5166
H6 -4.613 2.0091 -4.0163

H7 4.0754 1.074 -4.1093
H8 5.4152 2.5611 -3.4147
H9 7.3228 2.5645 -2.4968
H10 8.6972 1.0774 -1.8906
H11 4.0334 1.2385 3.053
H12 5.8741 2.5418 2.5469
H13 6.0434 3.6223 0.4815
H14 4.3761 3.3197 -1.0832
H15 2.5187 2.0091 -0.5836
H16 -3.0563 1.074 -0.4906
H17 -1.7165 2.5611 -1.1851
H18 0.1911 2.5645 -2.1031
H19 1.5655 1.0774 -2.7092
H20 4.0334 -1.2385 3.053
H21 5.8741 -2.5418 2.5469
H22 6.0434 -3.6223 0.4815
H23 4.3761 -3.3197 -1.0832
H24 2.5187 -2.0091 -0.5836
H25 -3.0563 -1.0741 -0.4906
H26 -1.7165 -2.5611 -1.1851
H27 0.1911 -2.5646 -2.1031
H28 -6.7034 -2.325 -6.7921
H29 -3.0983 -1.2385 -7.6529
H30 -1.2576 -2.5418 -7.1467
H31 -1.0883 -3.6223 -5.0813
H32 -2.7556 -3.3197 -3.5166
H33 -4.613 -2.0091 -4.0163
H34 4.0754 -1.0741 -4.1093
H35 5.4152 -2.5611 -3.4147
H36 7.3228 -2.5646 -2.4968
H37 8.6972 -1.0774 -1.8906
H38 2.8327 0.6908 -8.2888
H39 1.2997 0.2409 -8.5498
H40 2.0296 -1.8226 -7.8419
H41 3.2901 -1.1924 -7.0616
H42 1.0953 1.8976 -6.7924
H43 2.3485 1.2678 -5.989
H44 2.11 -1.0682 -5.1385
H45 0.9402 -1.9034 -5.8634
H46 9.9644 -0.6909 3.6889
H47 8.4314 -0.2409 3.95
H48 9.1613 1.8226 3.2421
H49 10.4219 1.1924 2.4617
H50 8.227 -1.8976 2.1925

H51 9.4802 -1.2679 1.3891

H52 9.2418 1.0681 0.5386

H53 8.0719 1.9034 1.2635

H54 1.5655 -1.0774 -2.7092

H55 0.9277 3.3011 2.8737

H56 0.3905 3.3163 1.3663

H57 -0.6122 2.9891 2.5699

H58 -0.6648 -3.0117 2.1848

H59 0.7428 -3.3805 1.5187

H60 0.6278 -3.2147 3.1063

H61 -10.1881 1.074 -4.1093

H62 -8.8483 2.5611 -3.4147

H63 7.5601 2.3249 -6.7921

H64 9.738 1.1956 -6.0301

H65 -10.1881 -1.0741 -4.1093

H66 -8.8483 -2.5611 -3.4147

H67 7.5601 -2.325 -6.7921

H68 9.738 -1.1956 -6.0301

H69 -5.5663 -1.0774 -1.8906

H70 -5.5663 1.0774 -1.8906

H71 -6.9407 -2.5646 -2.4968

H72 -6.9407 2.5645 -2.4968

V.3.2 Input structure for **2-Er** in Angstrom.

Er 5.3975 13.2423 14.8447

C 7.7986 12.8503 15.3899

H 8.5443 12.5214 14.9027

C 7.0849 11.8386 16.0266

H 7.4761 10.9822 15.9036

C 5.9306 11.7945 16.8005

H 5.6927 10.9090 17.0504

C 5.0397 12.7462 17.2951

H 4.3414 12.3536 17.8040

C 4.9299 14.1251 17.2265

H 4.1868 14.4558 17.7194

C 5.6500 15.1386 16.6010

H 5.2785 16.0001 16.7506

C 6.7891 15.1757 15.8080

H 7.0211 16.0589 15.5488

C 7.6703 14.2293 15.3038

H 8.3511 14.6211 14.7703

Sn 6.0264 13.2423 11.8124

Si	4.7723	16.3834	12.3135
C	2.7056	16.1591	10.3796
H	2.4030	16.2833	9.4555
H	2.0783	16.5989	10.9905
H	2.7466	15.2025	10.5846
C	5.0454	16.2192	9.4749
H	4.7255	16.4844	8.5872
H	5.0659	15.2412	9.5356
H	5.9471	16.5746	9.6171
C	4.0999	16.7789	10.5472
C	6.5775	16.8160	12.3215
H	6.6981	17.7230	11.9724
H	7.0675	16.1806	11.7586
H	6.9197	16.7702	13.2390
C	3.9427	17.6246	13.4688
H	4.2357	18.5309	13.2392
H	4.1926	17.4251	14.3952
H	2.9699	17.5607	13.3716
C	4.0032	18.3256	10.3637
H	3.7599	18.5309	9.4366
H	4.8693	18.7347	10.5718
H	3.3195	18.6848	10.9674
Si	5.1499	10.0377	12.6438
C	4.9312	7.8130	10.8679
H	4.7589	7.4761	9.9644
H	4.3244	7.3744	11.5012
H	5.8586	7.6204	11.1180
C	5.6134	9.9247	9.8196
H	5.3692	9.5527	8.9461
H	6.5450	9.6986	10.0220
H	5.5096	10.8986	9.8004
C	4.6954	9.3332	10.9030
C	6.9875	9.8187	12.7763
H	7.2329	8.9200	12.4719
H	7.2639	9.9332	13.7102
H	7.4374	10.4869	12.2188
C	4.3897	8.8653	13.9092
H	4.7997	7.9795	13.8220
H	3.4247	8.7951	13.7511

H	4.5482	9.2114	14.8120
C	3.2115	9.6510	10.5552
H	2.9727	9.2147	9.7104
H	3.0977	10.6204	10.4650
H	2.6297	9.3189	11.2701
C	2.2158	15.3611	14.9033
H	2.9617	15.3658	15.4868
C	4.6391	11.7751	12.8385
C	1.0691	16.1309	15.2527
H	1.0724	16.6601	16.0420
C	4.6756	14.5471	12.6614
C	-0.0526	15.3099	13.3507
H	-0.8275	15.2762	12.8027
C	-0.0379	16.1026	14.4597
H	-0.7953	16.6326	14.6763
C	2.3513	11.6638	13.8342
C	1.3033	11.2895	12.9805
H	1.2864	11.5948	12.0815
C	1.0682	14.5453	13.0204
H	1.0118	13.9479	12.2833
C	3.4728	12.4478	13.2917
C	0.2890	10.4685	13.4656
H	-0.4260	10.2193	12.8910
C	2.2663	14.6230	13.7225
C	1.3611	10.3449	15.5750
H	1.4002	10.0174	16.4656
C	3.5099	13.8779	13.2279
C	2.3664	11.1588	15.1091
H	3.0905	11.3626	15.6843
C	0.3060	10.0059	14.7788
H	-0.4014	9.4666	15.1124

V.3.3 Input structure for **2-Er^{TMS}** in Angstrom.

Er	5.3975	13.2423	14.8447
C	7.7986	12.8503	15.3899
H	8.5443	12.5214	14.9027
C	7.0849	11.8386	16.0266
H	7.4761	10.9822	15.9036
C	5.9306	11.7945	16.8005

H	5.6927	10.9090	17.0504
C	5.0397	12.7462	17.2951
H	4.3414	12.3536	17.8040
C	4.9299	14.1251	17.2265
H	4.1868	14.4558	17.7194
C	5.6500	15.1386	16.6010
H	5.2785	16.0001	16.7506
C	6.7891	15.1757	15.8080
H	7.0211	16.0589	15.5488
C	7.6703	14.2293	15.3038
H	8.3511	14.6211	14.7703
Sn	6.0264	13.2423	11.8124
Si	4.7723	16.3834	12.3135
C	4.1176	16.7685	10.5938
C	6.5775	16.8160	12.3215
H	6.6981	17.7230	11.9724
H	7.0675	16.1806	11.7586
H	6.9197	16.7702	13.2390
C	3.9427	17.6246	13.4688
H	4.2357	18.5309	13.2392
H	4.1926	17.4251	14.3952
H	2.9699	17.5607	13.3716
Si	5.1499	10.0377	12.6438
C	4.7077	9.3522	10.9500
C	6.9875	9.8187	12.7763
H	7.2329	8.9200	12.4719
H	7.2639	9.9332	13.7102
H	7.4374	10.4869	12.2188
C	4.3897	8.8653	13.9092
H	4.7997	7.9795	13.8220
H	3.4247	8.7951	13.7511
H	4.5482	9.2114	14.8120
C	2.2158	15.3611	14.9033
H	2.9617	15.3658	15.4868
C	4.6391	11.7751	12.8385
C	1.0691	16.1309	15.2527
H	1.0724	16.6601	16.0420
C	4.6756	14.5471	12.6614
C	-0.0526	15.3099	13.3507

H	-0.8275	15.2762	12.8027
C	-0.0379	16.1026	14.4597
H	-0.7953	16.6326	14.6763
C	2.3513	11.6638	13.8342
C	1.3033	11.2895	12.9805
H	1.2864	11.5948	12.0815
C	1.0682	14.5453	13.0204
H	1.0118	13.9479	12.2833
C	3.4728	12.4478	13.2917
C	0.2890	10.4685	13.4656
H	-0.4260	10.2193	12.8910
C	2.2663	14.6230	13.7225
C	1.3611	10.3449	15.5750
H	1.4002	10.0174	16.4656
C	3.5099	13.8779	13.2279
C	2.3664	11.1588	15.1091
H	3.0905	11.3626	15.6843
C	0.3060	10.0059	14.7788
H	-0.4014	9.4666	15.1124
H	4.2451	17.7209	10.4014
H	4.6036	16.2336	9.9319
H	3.1628	16.5516	10.5516
H	5.1849	9.8568	10.2586
H	4.9658	8.4081	10.9021
H	3.7418	9.4345	10.8063

V.3.4 Input structure for **2-Er^{tBu}** in Angstrom.

Er	5.3975	13.2423	14.8447
C	7.7986	12.8503	15.3899
H	8.5443	12.5214	14.9027
C	7.0849	11.8386	16.0266
H	7.4761	10.9822	15.9036
C	5.9306	11.7945	16.8005
H	5.6927	10.9090	17.0504
C	5.0397	12.7462	17.2951
H	4.3414	12.3536	17.8040
C	4.9299	14.1251	17.2265
H	4.1868	14.4558	17.7194
C	5.6500	15.1386	16.6010

H	5.2785	16.0001	16.7506
C	6.7891	15.1757	15.8080
H	7.0211	16.0589	15.5488
C	7.6703	14.2293	15.3038
H	8.3511	14.6211	14.7703
Sn	6.0264	13.2423	11.8124
C	4.7547	16.0484	12.3770
C	5.0682	10.3156	12.6750
C	2.2158	15.3611	14.9033
H	2.9617	15.3658	15.4868
C	4.6391	11.7751	12.8385
C	1.0691	16.1309	15.2527
H	1.0724	16.6601	16.0420
C	4.6756	14.5471	12.6614
C	-0.0526	15.3099	13.3507
H	-0.8275	15.2762	12.8027
C	-0.0379	16.1026	14.4597
H	-0.7953	16.6326	14.6763
C	2.3513	11.6638	13.8342
C	1.3033	11.2895	12.9805
H	1.2864	11.5948	12.0815
C	1.0682	14.5453	13.0204
H	1.0118	13.9479	12.2833
C	3.4728	12.4478	13.2917
C	0.2890	10.4685	13.4656
H	-0.4260	10.2193	12.8910
C	2.2663	14.6230	13.7225
C	1.3611	10.3449	15.5750
H	1.4002	10.0174	16.4656
C	3.5099	13.8779	13.2279
C	2.3664	11.1588	15.1091
H	3.0905	11.3626	15.6843
C	0.3060	10.0059	14.7788
H	-0.4014	9.4666	15.1124
C	3.9161	16.8058	13.4040
C	6.2080	16.5078	12.4680
C	4.2188	16.3297	10.9751
C	6.5906	10.2398	12.5846
C	4.5887	9.5063	13.8778

C 4.4535 9.7443 11.3993
H 2.9833 16.5109 13.3456
H 4.2600 16.6253 14.3037
H 3.9667 17.7674 13.2218
H 6.5520 16.3272 13.3678
H 6.7462 16.0217 11.8089
H 6.2587 17.4694 12.2859
H 4.7570 15.8436 10.3160
H 3.2860 16.0348 10.9166
H 4.2694 17.2913 10.7929
H 6.8983 10.7592 11.8126
H 6.9851 10.6065 13.4033
H 6.8655 9.3049 12.4798
H 4.9832 9.8730 14.6965
H 3.6116 9.5550 13.9358
H 4.8636 8.5715 13.7730
H 3.4764 9.7930 11.4572
H 4.7612 10.2637 10.6273
H 4.7283 8.8094 11.2945

V.3.4 Input structure for **2-Er^{tBu}** in Angstrom.

Er 5.3975 13.2423 14.8447
C 7.7986 12.8503 15.3899
H 8.5443 12.5214 14.9027
C 7.0849 11.8386 16.0266
H 7.4761 10.9822 15.9036
C 5.9306 11.7945 16.8005
H 5.6927 10.9090 17.0504
C 5.0397 12.7462 17.2951
H 4.3414 12.3536 17.8040
C 4.9299 14.1251 17.2265
H 4.1868 14.4558 17.7194
C 5.6500 15.1386 16.6010
H 5.2785 16.0001 16.7506
C 6.7891 15.1757 15.8080
H 7.0211 16.0589 15.5488
C 7.6703 14.2293 15.3038
H 8.3511 14.6211 14.7703
Sn 6.0264 13.2423 11.8124

C	4.7544	16.0434	12.3779
C	5.0668	10.3204	12.6755
C	2.2158	15.3611	14.9033
H	2.9617	15.3658	15.4868
C	4.6391	11.7751	12.8385
C	1.0691	16.1309	15.2527
H	1.0724	16.6601	16.0420
C	4.6756	14.5471	12.6614
C	-0.0526	15.3099	13.3507
H	-0.8275	15.2762	12.8027
C	-0.0379	16.1026	14.4597
H	-0.7953	16.6326	14.6763
C	2.3513	11.6638	13.8342
C	1.3033	11.2895	12.9805
H	1.2864	11.5948	12.0815
C	1.0682	14.5453	13.0204
H	1.0118	13.9479	12.2833
C	3.4728	12.4478	13.2917
C	0.2890	10.4685	13.4656
H	-0.4260	10.2193	12.8910
C	2.2663	14.6230	13.7225
C	1.3611	10.3449	15.5750
H	1.4002	10.0174	16.4656
C	3.5099	13.8779	13.2279
C	2.3664	11.1588	15.1091
H	3.0905	11.3626	15.6843
C	0.3060	10.0059	14.7788
H	-0.4014	9.4666	15.1124
H	4.2162	16.5296	13.0370
H	5.6872	16.3383	12.4363
H	4.4105	16.2240	11.4782
H	6.0439	10.2717	12.6175
H	4.7591	9.8010	13.4474
H	4.6723	9.9537	11.8568

VI. Supplementary References

1. Sheldrick, G.M. SHELXT– Integrated space-group and crystal-structure determination. *Acta Crystallographica Section A Foundations and Advances* **71**, 3-8 (2015).
2. Sheldrick, G.M. A short history of SHELX. *Acta Crystallogr A* **64**, 112-122 (2008).
3. Dolomanov, O.V., Bourhis, L.J., Gildea, R.J., Howard, J.A.K. & Puschmann, H. OLEX2: a complete structure solution, refinement and analysis program. *J. Appl. Crystallogr.* **42**, 339-341 (2009).
4. De, S., Mondal, A., Giblin, S.R. & Layfield, R.A. Bimetallic Synergy Enables Silole Insertion into THF and the Synthesis of Erbium Single-Molecule Magnets. *Angew. Chem. Int. Ed.* **63**, e202317678 (2024).
5. Li Manni, G. et al. The OpenMolcas Web: A Community-Driven Approach to Advancing Computational Chemistry. *J. Chem. Theory Comput.* **19**, 6933-6991 (2023).
