# Supplementary material for: Magnetic properties of monomeric and polymeric stannolediide yttrium and erbium complexes
Source: Commun Chem. 2025 Nov 21;8:368. doi: 10.1038/s42004-025-01797-4 (PMC12638953; doi:10.1038/s42004-025-01797-4)
Supplement: Supplementary file 2 — Description of Additional Supplementary Files [file 42004_2025_1797_MOESM2_ESM.pdf]

Description of Additional Supplementary Files:

File: Supplementary Data 1

Description: cif file of compound **1-Y**

File: Supplementary Data 2

Description: cif file of compound **1-Er**

File: Supplementary Data 3

Description: cif file of compound **2-Y**

File: Supplementary Data 4

Description: cif file of compound **2-Er**
